# Poor quality vital anti-malarials in Africa - an urgent neglected public health priority

**DOI:** 10.1186/1475-2875-10-352

**Published:** 2011-12-13

**Authors:** Paul N Newton, Michael D Green, Dallas C Mildenhall, Aline Plançon, Henry Nettey, Leonard Nyadong, Dana M Hostetler, Isabel Swamidoss, Glenn A Harris, Kristen Powell, Ans E Timmermans, Abdinasir A Amin, Stephen K Opuni, Serge Barbereau, Claude Faurant, Ray CW Soong, Kevin Faure, Jonarthan Thevanayagam, Peter Fernandes, Harparkash Kaur, Brian Angus, Kasia Stepniewska, Philippe J Guerin, Facundo M Fernández

**Affiliations:** 1 Wellcome Trust-Mahosot Hospital-Oxford University Tropical Medicine Research Collaboration, Microbiology Laboratory, Mahosot Hospital, Vientiane, Lao PDR; 2 Centre for Tropical Medicine, Nuffield Department of Medicine, University of Oxford, Churchill Hospital, Oxford, OX3 7LJ, UK; 3 Department of Infectious and Tropical Diseases, London School of Hygiene and Tropical Medicine, WC1E 7HT, UK; 4 WorldWide Antimalarial Resistance Network, Churchill Hospital, University of Oxford, Oxford, OX3 7LJ, UK; 5 Division of Parasitic Diseases and Malaria, Center for Global Health, Centers for Disease Control and Prevention, Atlanta, GA, 30329, USA; 6 GNS Science, Lower Hutt, 5040, New Zealand; 7 International Criminal Police Organization (INTERPOL), Lyon, 69006, France; 8 School of Chemistry and Biochemistry, Georgia Institute of Technology, Atlanta, GA, 30332-0400, USA; 9 Department of Immunology and Medicine, Armed Forces Research Institute of Medical Sciences (AFRIMS), Bangkok, 10400, Thailand; 10 Population Services International, Malaria and Child Survival Department, School Lane, Westlands, P.O. Box 14355-00800, Nairobi, Kenya; 11 Food and Drugs Board, P O Box CT 2783, Cantonments-Accra, Ghana; 12 Réseau Médicaments et Développment (ReMeD), 35, rue Daviel, 75013 Paris 13 France; 13 East West Pharmaceuticals, 189 rue Grande, Fontainebleau, 77300, France; 14 Mahidol Oxford Research Unit, Faculty of Tropical Medicine, Mahidol University, Bangkok, 10400, Thailand

## Abstract

**Background:**

*Plasmodium falciparum *malaria remains a major public health problem. A vital component of malaria control rests on the availability of good quality artemisinin-derivative based combination therapy (ACT) at the correct dose. However, there are increasing reports of poor quality anti-malarials in Africa.

**Methods:**

Seven collections of artemisinin derivative monotherapies, ACT and halofantrine anti-malarials of suspicious quality were collected in 2002/10 in eleven African countries and in Asia en route to Africa. Packaging, chemical composition (high performance liquid chromatography, direct ionization mass spectrometry, X-ray diffractometry, stable isotope analysis) and botanical investigations were performed.

**Results:**

Counterfeit artesunate containing chloroquine, counterfeit dihydroartemisinin (DHA) containing paracetamol (acetaminophen), counterfeit DHA-piperaquine containing sildenafil, counterfeit artemether-lumefantrine containing pyrimethamine, counterfeit halofantrine containing artemisinin, and substandard/counterfeit or degraded artesunate and artesunate+amodiaquine in eight countries are described. Pollen analysis was consistent with manufacture of counterfeits in eastern Asia. These data do not allow estimation of the frequency of poor quality anti-malarials in Africa.

**Conclusions:**

Criminals are producing diverse harmful anti-malarial counterfeits with important public health consequences. The presence of artesunate monotherapy, substandard and/or degraded and counterfeit medicines containing sub-therapeutic amounts of unexpected anti-malarials will engender drug resistance. With the threatening spread of artemisinin resistance to Africa, much greater investment is required to ensure the quality of ACTs and removal of artemisinin monotherapies. The International Health Regulations may need to be invoked to counter these serious public health problems.

## Background

*Plasmodium falciparum *malaria remains a major public health problem in much of the world, despite decades of interventions [1]. The tragedy remains that many more malaria patients would survive if they had timely access to good quality, affordable and efficacious medicines. With the implementation of pivotal artemisinin-based combination therapy (ACT) throughout malarious Africa and attempts to make it accessible and affordable, hope of controlling malaria has been rekindled [2,3]. Of 42 African countries with *P. falciparum *malaria, ACT is now national policy in 40 (95%)[4].

A diverse range of important problems reduce ACT effectiveness, including inaccessibility, poor prescribing, poor adherence and poor medicine quality. There are two main categories of poor quality medicine. Counterfeit (or falsified, spurious) medicines (i.e. '*deliberately and fraudulently mislabelled with respect to identity and/or source*;[5,6]) and substandard medicines (i.e. '*genuine medicines produced by manufacturers authorized....which do not meet quality specifications set for them by national standards'*;[6-8]). Substandard medicines frequently, and counterfeits occasionally, contain sub-therapeutic amounts of active pharmaceutical ingredients (API) and/or may show suboptimal release of API (dissolution), exposing parasites to sub-lethal concentrations of API(s) [8-10]. However, the percentage API in genuine medicines may also be reduced after manufacture if they are degraded by extremes of temperature and humidity [11].

Substandard and counterfeit anti-malarials were major problems in pre-ACT Africa. Those medicines with subtherapeutic amounts of API are likely to have contributed to the spread of resistance to previous generations of anti-malarials, such as sulphadoxine-pyrimethamine (SP) and chloroquine [10,12,13](Additional file 1). With evidence that SP and chloroquine-resistant *P. falciparum *entered Africa from SE Asia [14,15], the recent descriptions of artesunate resistance there [16] suggests that, in addition to major local repercussions, it is very likely to spread to Africa.

**Figure 1:**
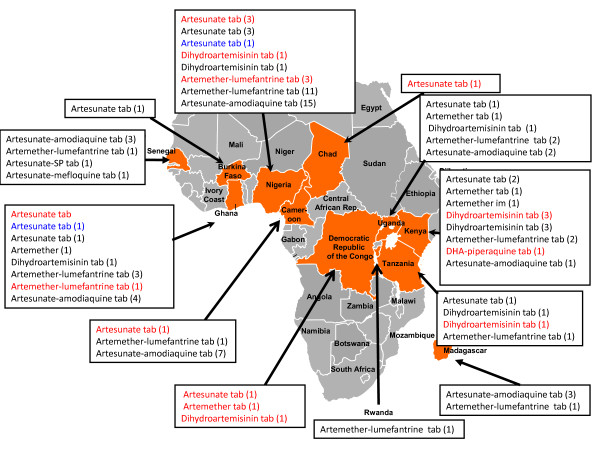
**Map of Africa with reports of poor quality artemisinin derivatives and ACT to October 2011**. Red = counterfeit, blue = substandard, black = poor quality (ie uncertain whether substandard or counterfeit).

**Figure 2:**
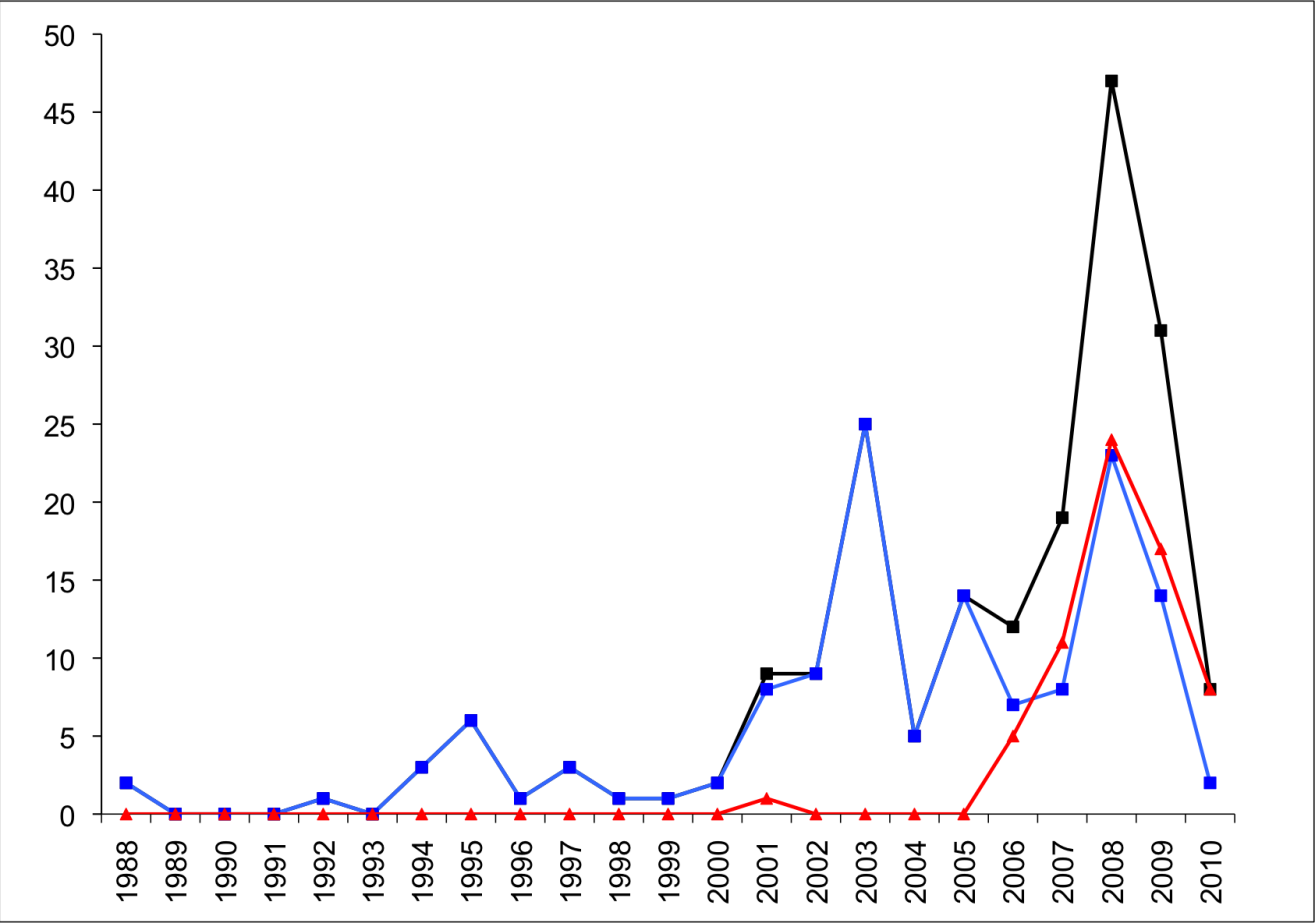
**Frequency of reports of poor quality anti-malarials in Africa per year 1988-2010, for all anti-malarials (black), non-artemisinin derivatives (blue) and artemisinin derivative montherapies and ACTs (red)**. Each poor quality medicine type (by API or brand name per country per report) included. Reports of more than one poor quality medicine of the same brand name/API/formulation per paper classed as one report. The fall in frequency in 2010 is probably an artifact of investigations-up to October 2011 there have already been 64 reports of poor quality anti-malarial types in Africa. See Additional file 1.

There are increasing reports of poor quality artemisinin monotherapies in Africa (Figures 1, 2, Additional file 1). However, monotherapies, even when of good quality, should be replaced by ACTs [1]. Although poor quality ACT has yet to be reported in Asia, there has been an alarming increase in reports of poor quality ACTs in Africa [6,17-23](Additional file 1, Figure 2). Poor ACT quality, along with poor prescribing and poor adherence, would provide a fertile environment for the spread of artemisinin-resistant parasites. This would destroy the renewed hope for malaria control in Africa and killing many patients who would otherwise survive. Therefore, the authors offered to analyse anti-malarial medicines of suspicious quality in sub-Saharan Africa via meetings, INTERPOL and the Counterfeit Drug Forensic Investigation Network (CODFIN [24]).

## Methods

Analyses of packaging, chemistry and botany were performed blindly. The results have been reported using the MEDQUARG guidelines where possible [25]. Samples were stored at +4°C for between 1 month and 6 years before analysis. Counterfeit, substandard and degraded were defined based on the above definitions, with packaging analysis, in comparison to authentic samples, as the crucial evidence for the distinction between counterfeit and substandard/degraded. This was performed without any intellectual property considerations. When data did not allow such classification but the sample contained API% outside reference ranges, such medicines are referred to as 'poor quality'.

### Physical appearance

The physical appearance and text on packaging were examined and compared with known genuine samples when available [26]. Nineteen companies were asked for genuine samples of their products and 6 (32%) responded. DigitalColorMeter (v3.4.1, Apple Inc.) was used to measure the percentage of red (R), green (G) and blue (B) pixels at predefined points on the packaging.

### Chemical investigations

Sample API was quantified using a modified high performance liquid chromatography (HPLC) method with photo diode array detection [27]. Samples were also screened by two direct ionization mass spectrometry (MS) methods: Direct Analysis in Real Time (DART) and Desorption Electrospray Ionization (DESI) in both conventional and reactive modes [28,29]. Select samples were also analysed by X-ray diffractometry (XRD; X'Pert Pro, Philips, Almelo) and isotope ratio MS to determine the mineral composition [26]. GlaxoSmithKline plc (GSK) performed analysis of 'halofantrine' samples using Fourier Transform Infra Red spectroscopy (FTIR), verified when necessary by HPLC with electrospray ionization and mass spectrometry detection (Eckers & Wolffe pers. comm.)(Additional file 2).

A sample was defined as one dosage unit-such as a blister or blisters in one packet (if present) or one bottle. The %API range, relative to the stated dosage, allowed on HPLC analysis was 90-110%. An important issue, barely addressed, is that in work such as this, the available sample size of dosage units (such as one blister) is considerably less than that required in testing described in pharmacopoeias [e.g. [30]]. Testing for uniformity commonly requires that if one tablet in a sample contains < 75% or > 125% of API relative to the stated dosage, the sample fails the assay [30]. Tablets within this range may represent a Type II error of accepting a sample when it is poor quality. Ignoring the variability in chemical assays, assuming that tablet API% in an "acceptable" population is normally distributed with standard deviation of < 5% (to ensure that all tablets are within 75-125% range) there are, at most, chances of 1.3/1,000, 2.3/1,000 and 0.5/1,000 of finding a tablet with 75%-85%, 75%-90% and 75%-90% API, respectively. Therefore, for tablets with API > 75%, taking a pragmatic approach that a sample fails if more than 1 dosage unit falls outside the 90-110% range, carries a relatively small chance of rejecting a compliant sample or Type I error.

### Biological investigations

Tablet samples were analysed for pollen/spores [26], which could be indicative of either the place of manufacture, the source of the individual ingredients or both, and be influenced by wind dispersal, seasonality and transplantation beyond their known natural range.

## Results

Seven sets of anti-malarials, of ten different types, were collected in eleven African countries 2002-2010 (Table 1, Additional file 2). The results were reported to the appropriate national Medicines Regulatory Agency (MRA) and the company as stated on the packaging.

**Table 1:** Summary of case reports of poor anti-malarial medicine quality in Africa.

Medicine Country	Classification	Stated Manufacturer	Chemistry/Pollen	Packaging/Notes
'Artesunate' 50 mg tablets Cameroon	Counterfeit	'Mekophar Chemical Pharmaceutical Joint-Stock Company' Marketed by: 'Neros Pharmaceuticals Ltd., Lagos Nigeria'	Contains chloroquine. No artesunate detected. Pollen grains of bulrush (*Typha angustifolia*). Pollen and chemistry suggests two different types	Collected because of suspicion of their low cost (1500 FCFA ~$3/pack of 12 tablets). Packet and leaflet colours and holograms differed and the counterfeit packets were heavier.

'Artesunate' 100 mg tablets DR Congo	Poor quality	'Marinate'. No manufacturers details on the packaging. Only 'GUJ/DRUGS/1407'	79 and 88% artesunate/tablet, but analysed 19 months after expiry	Probably labeled as made in Gujerat, India. Genuine packaging not available

'Artesunate' 100 mg tablets DR Congo	Counterfeit	Astrinate on one face of leaflet and Arinate on reverse Stated to be 'Manufactured for AT17 RUE POISSONNIERS 75018 PARIS'	88.5, 70.9 mg artesunate/tablet	No agent/company in Rue Poissonniers. 'Arinate' is the trade name of artesunate monotherapy from Dafra Pharma, Belgium.

'Artesunate' 50 mg tablets Ghana	Poor quality	'LEVER Artesunate' Stated to be manufactured by 'ADAMS PHARAMCEUTICAL (ANHUI) CO., LTD. ANHUI, CHINA Division of Sunflower Int'l Group'	artesunate 39.0, 43.0, 47.0, 47.0 mg/tablet	Unable to obtain sample of genuine packaging

'Dihydroartemisinin' 60 mg tablets Kenya	Counterfeit	'Jiaxing Nanhu Pharmaceutical Co. Ltd. Jiaxing City, under license of Beijing Holley-Cotec Pharmaceuticals (PR China)' with stated trade name 'Cotecxin'	No dihydroartemisinin detected. Chenopodiaceae pollen consistent with a source in SE China or in adjacent areas of SE and south Asia but not with Africa	The counterfeit tablet diameter and blister foil are larger and markedly lighter orange, respectively

'Halofantrine' 250 mg tablets Sierra Leone, Nigeria, Cameroon, DRC, Tanzania, Liberia, China	Counterfeit	'SmithKline Beecham Laboratoires Pharmacetiques'	One contained correct % halofantrine-fraudulent extension of expiry date. Artemisinin, acetaminophen, dipyrone, pyrimethamine wrong APIs. *Fagopyrum *(buckwheat) and *Sesamum *(sesame) pollen in counterfeits. Consistent (but does not prove) with seasonally arid source in southern China. *Betula *(birch) pollen grain and a *Stenochlaena *fern spore in other counterfeits suggesting E/SE Asia, inconsistent with India/Africa	12 counterfeit Halfan tablets were classified as Types A, B, C, D, & E. Type A contained artemisinin while B, C, D & E contained acetaminophen or no API detected

Halofantrine Syrup 30 ml bottle DR Congo	Counterfeit	'SmithKline Beecham Laboratoire Pharmaceutiques Esplanade Charles de Gaulle 92731 NANTERRE Cedex,'	No API detected	Came with a spoon, leaflet and packet, suggesting considerable investment in deception.

'Dihydroartemisinin-piperaquine' 40/320 mg tablets China	Counterfeit	'Zheijiang Holley Nanhu Pharmaceutical Group Ltd Under license of HolleyPharm' with stated trade name 'Duo-Cotecxin'	Sildenafil (Viagra; median (range) 10.4 (6.1-18.4) mg/tablet) detected in the matrix of the counterfeit tablets. *Cibotium *fern, widespread in SE and south Asia in both counterfeits and comparator genuine samples, suggesting that both manufactured in same region	Packaging with language errors with English and French (Franglais)

'Artemether-lumefantrine' 20/120 mg tablets Ghana	Counterfeit	'Beijing Novartis Pharma Ltd, Beijing, China'	No artemether or lumefantrine detected. Contain pyrimethamine (6.2-25 mg/tablet) and unidentified yellow pigment in counterfeits. Pollen in counterfeit samples consistent with manufacture in E/SE Asia but not in Africa or India	8 tablets/blister for counterfeits rather than 6 for genuine. Errors in German language spelling

'Artemether-lumefantrine' 20/120 mg tablets Cameroon	Counterfeit	'Beijing Novartis Pharma Ltd, Beijing, China'	No artemether or lumefantrine detected. Contained pyrimethamine and sulphadiazine	6 tablets/blister for counterfeits

Artesunate & amodiaquine co-blistered. 100/300 mg tablet Ghana	Poor quality	'Pharmanova Limited, Accra, Ghana. Manufactured by: Atlantic Pharmaceuticals Limited Accra, Ghana'	Artesunate 92.0, 103 mg/tablet and amodiaquine 237, 240 mg/tablet	Unable to obtain sample of genuine packaging

See Additional File 2 for details.

### Artesunate monotherapy

Six blisters of 'Mekophar' artesunate tablets were collected in 2007 in Bamenda, Cameroon. All contained chloroquine but no artesunate was detected by HPLC or MS. XRD and botanical analysis suggested that CAM S5/07 had a different source from the other counterfeits. Bulrush (*Typha angustifolia*) pollen was found in this specimen only, suggesting use of contaminated water or a source near swampland in East Asia or Africa.

**Figure 3:**
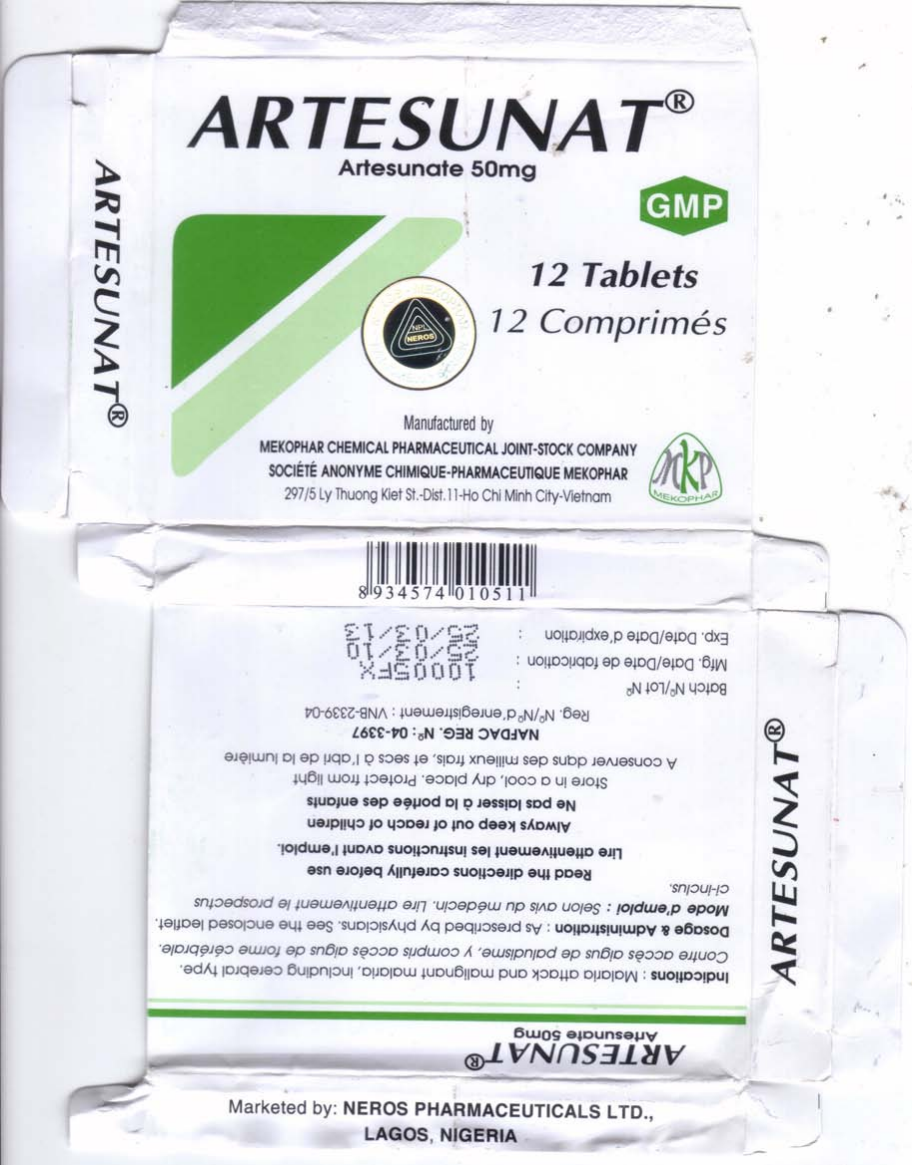
**Genuine artesunate 'Mekophar Chemical Pharmaceutical Joint-Stock Company' packet (Mek 10/03)**.

**Figure 4:**
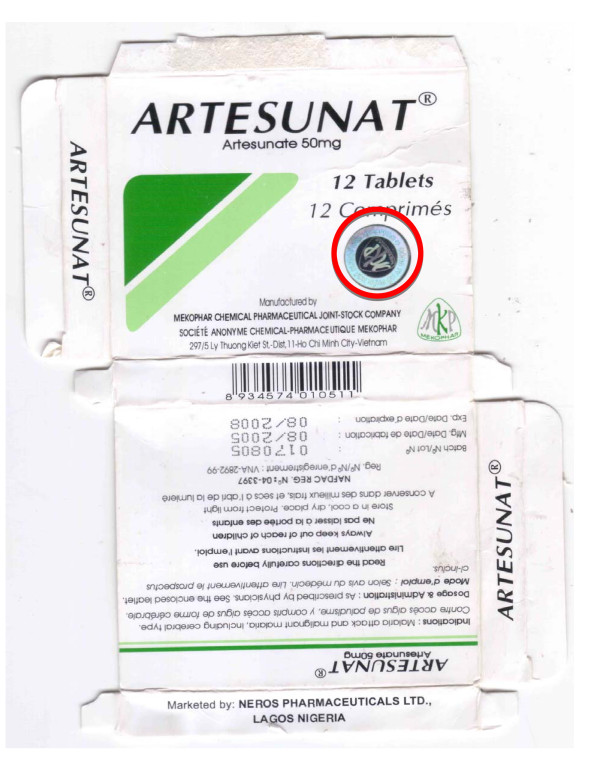
**Counterfeit artesunate packet labelled as made by 'Mekophar Chemical Pharmaceutical Joint-Stock Company' (Cam S5/07)**. Counterfeit hologram in red circle.

**Figure 5:**
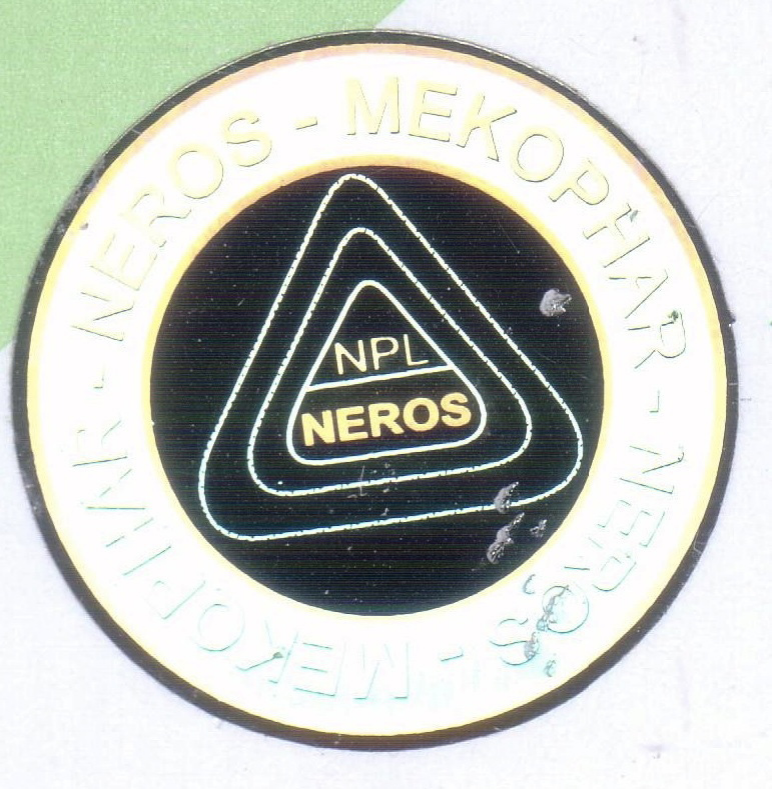
**Genuine artesunate with 'Mekophar Chemical Pharmaceutical Joint-Stock Company' and 'Neros' packet hologram (Mek 10/03)**.

**Figure 6:**
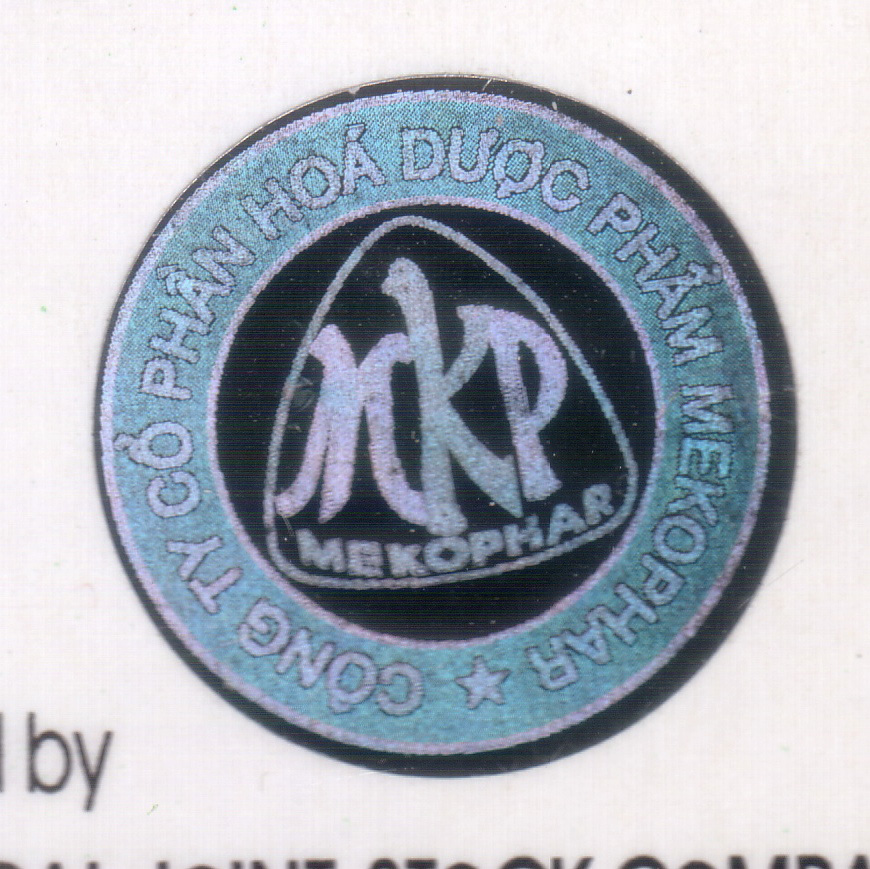
**Counterfeit hologram on packet labelled as made by 'Mekophar Chemical Pharmaceutical Joint-Stock Company' (Cam S5/07)**.

The counterfeits were printed with a Nigerian National Agency for Food and Drug Administration and Control (NAFDAC) registration number and a statement that they were marketed by 'Neros Pharmaceuticals Ltd., Lagos, Nigeria', which is the distributor of the genuine product. In comparison to genuine equivalents (Mek 10/03,10/05), the packaging colours and holograms differed and the counterfeit packets were heavier. The genuine hologram was introduced in response to counterfeits but the counterfeit hologram is not similar to those used by Mekophar (Figures 3, 4, 5 and 6, Additional file 3).

**Figure 7:**
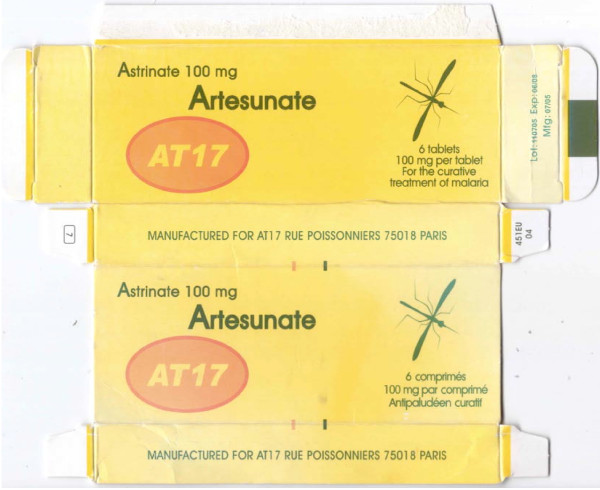
**Counterfeit artesunate packet labelled as made by 'AT17'**. **(**DRC 07/01).

In addition, six and one samples of artesunate monotherapy were collected by AET and SB, respectively, in the Democratic Republic of Congo (DRC) in 2007/8. One sample (DRC 08/01) is poor quality with two tablets, stated to contain 100 mg artesunate/tablet, containing 79 and 88 mg artesunate as determined by HPLC. These tablets were analysed 19 months after the expiry date, which could account for the low %API. Since the packaging contained no manufacturer details, it was not possible to compare with genuine examples but casts doubt on its authenticity. DRC 07/01 (Figure 7) was counterfeit, stated to be manufactured for 'AT17' in Paris. No evidence was found that this company exists in France. Analysis of this sample showed that it contained 70.9-88.5 mg artesunate/tablet.

In ad hoc sampling in West Africa in 2008 (by JT & PF) of 13 artesunate monotherapies, one (Gh 08/15) had 2/4 tablets outside reference API% range. The authors were unable to obtain genuine samples of this product and it may be substandard, degraded or counterfeit. An additional six (46%) samples had one tablet with artesunate content < 90%, relative to the stated dose.

### Dihydroartemisinin (DHA) monotherapy

**Figure 8:**
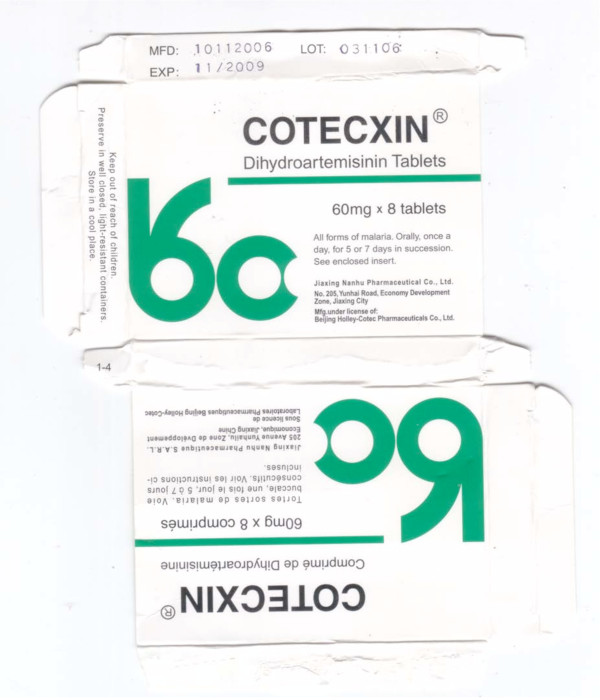
**Genuine dihydroartemisinin (DHA) made by 'Jiaxing Nanhu Pharmaceutical Co. Ltd.' packet (Ken 07/01)**.

**Figure 9:**
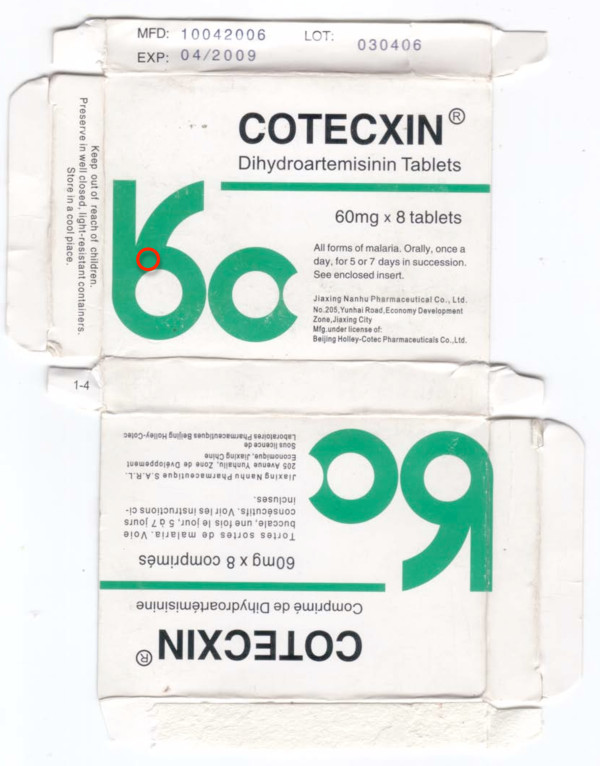
**Counterfeit DHA packet labelled as made by 'Jiaxing Nanhu Pharmaceutical Co. Ltd.' (Ken 07/02)**. Different shades of green, e.g. in red circle, from Ken 07/01.

Two samples labelled as dihydroartemisinin tablets (Cotecxin™) were collected in Kenya in 2007 [23,31]. HPLC analysis gave a DHA content of Kenya 07/01 of 101%, relative to the stated dose whilst no DHA was detected in Kenya 07/02. Only three pollen grains were identified in Kenya 07/01, including *Hibiscus *species, which are cultivated over many tropical and temperate areas. Kenya 07/02 contained abundant brown fungal spores but only eight pollen grains including Chenopodiaceae pollen, consistent with a source in SE China or in adjacent areas of SE/south Asia but not with Africa. The packaging of Kenya 07/01 and 07/02 were extremely similar. The same differences in tablet size and blister colour as previously described were noted [23]. The counterfeit tablet diameter and blister foil are larger and markedly lighter orange, respectively, than genuine samples (Figures 8 &9, Additional file 4).

Of the three DHA samples collected during ad hoc sampling in West Africa, although relatively few tablets could be analysed, all had one tablet outside reference %API range. No differences were detected in the packaging of Gh 08/07 in comparison to samples from the authentic manufacturer. A sample of DHA collected in Nigeria was counterfeit [32] and was found to contain paracetamol (acetaminophen) by MS.

### Halofantrine monotherapy

**Figure 10:**
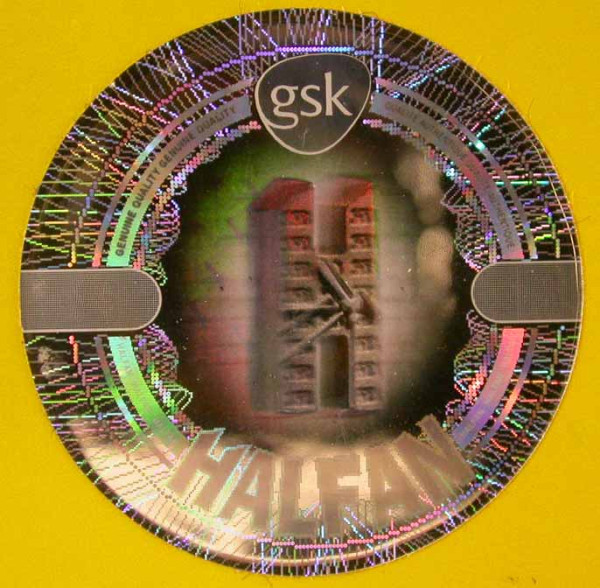
**Genuine halofantrine Halfan GlaxoSmithKline hologram**.

**Figure 11:**
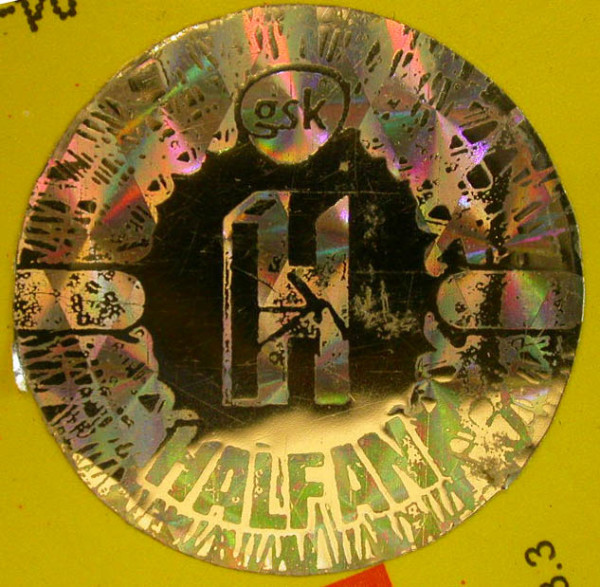
**Counterfeit 'Halfan 'hologram labelled as made by 'GSK' (4040 & 4023)**. GSK = GlaxoSmithKline.

**Figure 12:**
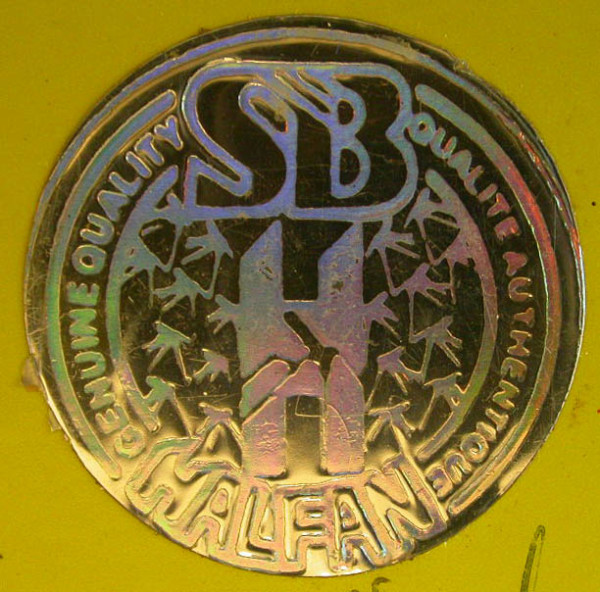
**Counterfeit 'Halfan' hologram labelled as made by 'SB' (4024)**. SB = SmithKline Beecham.

**Figure 13:**
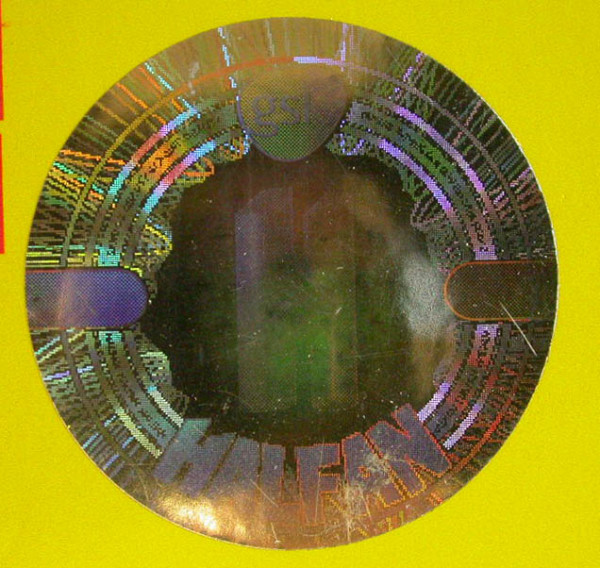
**Counterfeit 'Halfan' hologram labeled as made by 'GSK' (5070, 5312)**. GSK = GlaxoSmithKline.

Eighteen samples of Halfan™ (halofantrine) tablets, provided by GSK, included two genuine samples and 16 counterfeit examples collected in Sierra Leone (2), Nigeria (9), Cameroon (1), DRC (1), Tanzania (1), Liberia (1) and China (1) in 2002/2007. Of the counterfeit samples, four (25%) contained artemisinin, seven (44%) contained acetaminophen, one (6%) contained dipyrone, one (6%) contained pyrimethamine, one (6%) contained halofantrine and no API was detected in two (13%). Those containing artemisinin were found in Sierra Leone and Nigeria whilst those containing acetaminophen were found in Nigeria, Cameroon, Liberia and China. The median (range) content/tablet of artemisinin was 38 (21-70) mg and 156 (< 1-317) mg for paracetamol. XRD of 4 counterfeits demonstrated diverse excipients with one sample containing only starch and organic compounds (as in genuine 'Halfan') and three containing a variety of combinations of talc, starch and calcite. The sample with only starch and organic compounds detected, contained no detectable pollen and was originally genuine, containing 250 mg halofantrine/tablet, but had manufacturer and expiry dates fraudulently extended by two years. Thus, pollen, packaging and XRD were able to correctly identify this form of counterfeiting by expiry date tampering. *Fagopyrum *(buckwheat) and *Sesamum *(sesame) pollen grains were found in one counterfeit halofantrine sample. Sesame grows in arid areas in India, SE Asia, China and northern Australia, whilst *Fagopyrum *grows in North America and China. The absence of wind blown grass pollen suggests a seasonally arid area, consistent with a source in southern China. Another sample contained a *Betula *(birch) pollen grain and a *Stenochlaena *fern spore, suggesting East/SE Asia as a source, inconsistent with origins in India or Africa. The packaging of 12 counterfeit Halfan tablets were classified by GSK into Types A, B, C, D, & E. Type A contained artemisinin while B, C, D & E contained acetaminophen or no API (Figures 10, 11, 12 and 13).

A sample of counterfeit halofantrine syrup, collected (by SB) in Kivu, DRC, in 2006 had no API detected. Nor did it contain sulphamethazine as previously reported from another counterfeit halofantrine syrup [33]. This came with a spoon, leaflet and packet, suggesting considerable investment in deception.

### Dihydroartemisinin-piperaquine ACT

Eight blisters of co-formulated dihydroartemisinin-piperaquine (Duo-Cotecxin) due to be shipped to Africa from China were provided by Holley-Cotec Pharmaceuticals, China in 2007. DHA and piperaquine were detected in the four genuine samples but not in the four counterfeit samples. Sildenafil (Viagra; median (range) 10.4 (6.1-18.4) mg/tablet) was detected in the matrix of the counterfeit tablets, not in the coating. Botanical analysis of both genuine and counterfeit samples revealed spores of the fern *Cibotium*, widespread in SE and south Asia, suggesting that both genuine and counterfeit manufacturers were in the same region.

**Figure 14:**
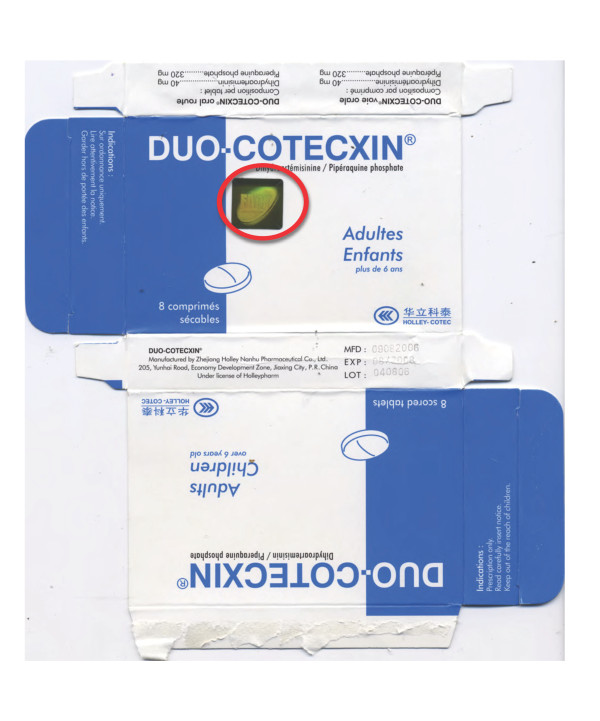
**Genuine 'Duo-Cotecxin' dihydroartemisinin-piperaquine packet made by 'Zheijiang Holley Nanhu Pharmaceutical Group Ltd Under license of Holleypharm' (China 07/14)**. Genuine hologram in red circle.

**Figure 15:**
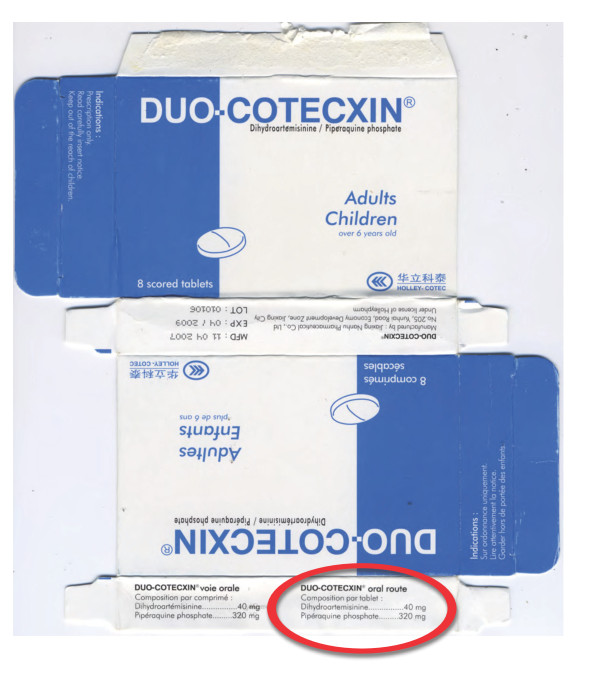
**Counterfeit dihydroartemisinin-piperaquine labeled as 'Duo-Cotecxin' made by 'Jiaxing Nanhu Pharmaceutical Group Ltd Under license of Holleypharm' (China 07/18)**. 'Franglais' in red circle.

The packaging of four counterfeits contained language errors with English and French combined ('Franglais') e.g. 'Composition par tablet' and 'Dihydroartemisinine' within the English text (Figures 14 &15, Additional file 5). In comparison to the genuine samples, the text was less clearly printed and lacked a hologram. The %B colour of the blue areas on the packet differed slightly from equivalent areas on the genuine packets (P = 0.02). Both genuine and fake contained blue-coated white tablets and the external tablet colours were similar in terms of RBG%. The fake tablets were significantly thicker and of significantly narrower diameter than the genuine tablets (P = 0.02).

### Artemether-lumefantrine ACT

The Food and Drug Board, Government of Ghana provided in 2009, via INTERPOL, two packets of 'Coartem' stated to be co-formulated artemether-lumefantrine (eight tablets/blister) and stated to be manufactured by 'Beijing Novartis Pharma Ltd'. Samples of genuine private sector Coartem with eight tablets/blister were not available and therefore a sample bought in Kenya was used as a comparator (Ken 06/01). Both Ghanaian samples contained no detectable artemether or lumefantrine but did contain pyrimethamine (6.2-25 mg/tablet). All tablets analysed were yellow, which in the case of genuine Coartem results from the presence of lumefantrine. An insoluble (in weak acid) yellow pigment was detected in both counterfeit samples but no amodiaquine, another yellow anti-malarial, was detected by HPLC or MS. The yellow pigment was adsorbed on a nylon filter membrane and eluted with alkaline methanol and had maximum absorbance at 425 nm but could not be identified by MS. The genuine Coartem samples contained *Dacrydium pierrei *pollen, a tree growing in southernmost China and in the mountains of northern SE Asia. Pollen in the counterfeit samples were consistent with manufacture in E/SE Asia but not in Africa or India.

**Figure 16:**
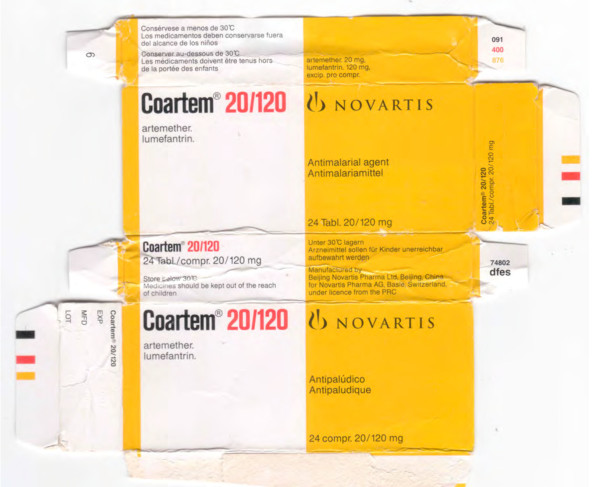
**Genuine artemether-lumefantrine 'Coartem' made by 'Beijing Novartis Pharma Ltd, Beijing, China for Novartis Pharma AG, Basle, Switzerland' (Ken 06/01)**.

**Figure 17:**
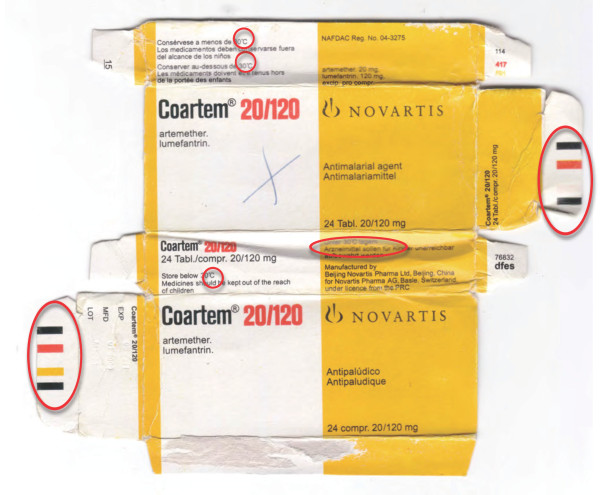
**Counterfeit 'Coartem' labelled as made by 'Beijing Novartis Pharma Ltd, Beijing, China for Novartis Pharma AG, Basle, Switzerland' (Gh 09/01)**. Differences from genuine sample (Figure 16) in red circles.

**Figure 18:**
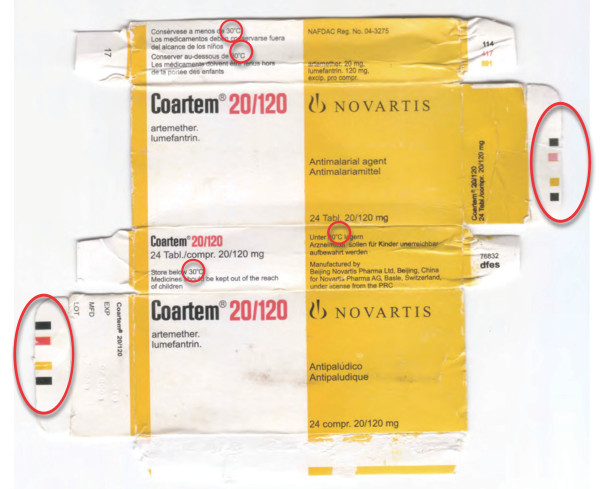
**Counterfeit 'Coartem' labelled as made by 'Beijing Novartis Pharma Ltd, Beijing, China for Novartis Pharma AG, Basle, Switzerland' (Gh 09/02)**. Differences from genuine sample (Figure 16) in red circles.

The packaging suggested that Gh 09/01 was counterfeit (Figures 16, 17 and 18, Additional file 6). The counterfeiters confused 'm' with 'rn' in 'lagern' (German for storing) and printed the packets with the word 'lagem' in error. Initially Gh 09/02 was thought to be genuine, but 8 tablets/blister and smudged codes on the blister implied that it was also counterfeit (Figure 18, Additional file 6), supported by the absence of APIs. The batch numbers of counterfeit 'Coartem' as released by the Food and Drug Board (FDB), Government of Ghana, were X0089 and M1200 [18,19], the same as reported here. Interestingly, it was reported that 'the fake CoArtem contains three strips, each with eight tablets and sold at GH 6.00 while the original one contains four strips with six tablets and sold at Gh 9.00'[18]. That eight tablets were present in each counterfeit Ghanaian blister is further evidence that they were counterfeit, as Novartis changed to six tablets/blister in 2007 whilst the samples were labelled as manufactured in 2008 and 2009.

Suspect Coartem from Cameroon were provided via Novartis and INTERPOL in 2010. These consisted of blisters of six tablets, labelled as made in 2008, containing no artemether-lumefantrine, but containing pyrimethamine and sulphadiazine. This suggests that the criminals producing them differed from the counterfeiters operating in Ghana or that they changed their packaging in response to changes made by Novartis.

### Artesunate+amodiaquine ACT

Of one co-blistered artesunate+amodiaquine collected in Ghana, 1/2 pairs of tablets (only 3 pairs per blister) analysed contained less than the reference range for artesunate and amodiaquine and may be counterfeit, substandard or degraded.

## Discussion

This wide diversity of different counterfeit and substandard anti-malarials from eight sub-Saharan African countries are cause of great concern. However, this study has important limitations, especially since it involved ad hoc collection and estimates of the frequency of poor quality anti-malarials cannot be deduced from these data. Tablet dissolution was not measured. The sampling method will bias towards finding counterfeit, rather than substandard, anti-malarials. It provides early warning, worrying for public health, as would a case series of new rapidly fatal epidemic influenza strains. However, that counterfeit formulations of ACT have been found at all is extremely alarming and will increase treatment failure, death and morbidity, yield covert unprotected monotherapy, increase the frequency of anti-malarial drug resistance and produce unexpected and clinically confusing adverse events. That 32% of artesunate and all DHA and DHA-piperaquine in the West Africa and DRC collections had one analysed tablet outside %API reference range is difficult to interpret. Analysis with larger numbers of tablets/sample is needed to understand the clinical implications.

There has been a dramatic rise in reports of poor quality non-artemisinin and artemisinin containing anti-malarials in Africa, suggesting an important worsening situation and/or an emerging interest (Figure 2)[34-36]. There are no data available that allow accurate estimation of the prevalence of poor quality anti-malarials in Africa, but enough information is available to know that it is a serious problem. Public health bodies should not wait for large-scale evidence based on random surveys to decide on interventions, as these data will take years to acquire. Unless action is taken quickly, poor ACT quality and profligate use of monotherapy (whether genuine or poor quality) will contribute to the failure of ACT. Although, the correlation between artemisinin resistance and poor drug quality has not been proven, modeling strongly suggests that underdosing is an important factor in the spread of *P. falciparum *drug resistance [13]. Poor quality anti-malarials, usually substandards, resulting in blood concentrations between those that kill resistant and sensitive parasites and those that kill only sensitive parasites will select for drug resistance. Counterfeits may also aid and abet this process by increasing the risk of hyperparasitaemia and recrudescence and the co-circulation of substandard and counterfeit medicines may be especially prone to engender drug resistance especially where patients 'shop' around when treatments fail.

The initial misclassification of a counterfeit artemether-lumefantrine as genuine emphasizes the importance of analysing both packaging and the pharmaceutical chemical composition and the importance of access to samples of the authenticated packaging, which was found to be difficult with only 32% companies responding. In addition, we may have classified counterfeit samples, containing correct %API, as genuine because we were unable to compare all samples with genuine packaging.

The discovery of unexpected APIs in counterfeit anti-malarials have important public health implications [37]. Unexpected pyrimethamine, especially if taken repeatedly, could give rise to clinically-confusing adverse effects, such as bone marrow suppression, rash and insomnia [38]. Depending on the background level of *P. falciparum *antifolate resistance [39,40], they may-at least initially-alleviate some malaria symptoms but are extremely unlikely to be curative. Pyrimethamine, in combination with sulphadoxine, is still used in sub-Saharan Africa, especially for Intermittent Preventive Treatment in pregnancy (IPTp) [4]. The presence of pyrimethamine as hidden monotherapy in counterfeits will engender the further spread of *P. falciparum *dihydrofolate reductase mutations in Africa, increasing therapeutic failure and reducing the useful life of SP for IPTp. Covert consumption of anti-malarials will also confuse our understanding of changes through time of the frequency of clinical failure and molecular markers of chloroquine and SP resistance [39-41]. The median concentration of sildenafil, a wrong API in DHA-piperaquine, was 10.4 mg/tablet, whilst the smallest dose/tablet of Viagra is 25 mg, suggesting that covert administration may cause unexpected penile erection and, as it is not usually taken by the acutely ill, unknown complications. The consequences could be severe especially as this drug is contraindicated in those with hypotension and myocardial ischaemia. In addition, patients may be exposed to dangerous drug interactions between covert consumption and other medicines patients may take, such as sildenafil with anti-HIV medication and chloroquine and pyrimethamine with anti-epileptics [38]. Pollen analyses of the counterfeit anti-malarials were consistent with an origin in eastern Asia, but do not prove this. In 2001 Guangzhou police arrested Nigerian and Chinese men for production of counterfeit halofantrine [42]. No evidence was found, from pollen analysis, of counterfeit pharmaceutical production in Africa. However, production facilities for counterfeit anti-malarial packaging have been seized in Nigeria [43].

What should be done [6,8,10,12,44]? Multiple parallel strategies are urgently needed to improve the quality of medicines people take and to ensure that they are available and taken at the recommended doses. The enormous investment in the development, evaluation and deployment of anti-malarials is wasted if the medicines that patients actually take are, due to criminality or carelessness, sub-therapeutic. Objective data on the epidemiology of poor quality anti-malarials are needed to allow quantification and mapping of the problem [40], the relative public health importance of counterfeit and substandard 'products', determine intervention prioritization and, through following changes through time, evaluate their effectiveness. Second, WHO estimated that 30% of countries have either 'no drug regulation or a capacity that hardly functions' [45,46] and presumably many of these are economically-poor and malarious. MRAs are keystones for crucial interventions to improve medicine quality and without them most interventions are doomed. There are only three countries with WHO pre-qualified Quality Control medicine analysis laboratories in the whole of malarious Africa [47]. Investment in African MRAs and quality-assured medicine quality laboratories would facilitate countries ability to regulate medicines. There is a danger of poor quality medicines use in clinical trials, likely to bias results and therefore mandatory testing of such medicines should be carried out, preferably at one of the WHO-prequalified laboratories. Third, increasing the reach of affordable good quality ACT will reduce mortality [2,3] and undercut the counterfeiters, by reducing their profit margins. Stockouts of ACT may encourage poor quality anti-malarial distribution. Fourth, artemisinin monotherapies are still very widely available in large quantities [37,48], despite appeals to restrict their use, and their removal where patients have access to ACT is a key intervention. Fifth, much more attention needs to be paid to substandard medicines, with inspection and facilitation of good quality production. Sixth, increased cooperation between MRAs, police, customs, malaria control programmes, pharmaceutical companies and international organizations is vital in countering the trade in counterfeit medicines. Seventh, new portable and rapid techniques, based on Raman and Near-Infrared spectroscopy, have been used in the screening of medicine quality and assisted in recent seizure of imported counterfeit ACT in Nigeria [21]. Although which technique is the most accurate and appropriate remains unclear, they could potentially empower drug inspectors in the screening of pharmacy stock for poor quality medicines. Eighth, we are woefully ignorant as to how best to tackle poor medicine quality in different situations and there has been a damaging lack of public health, civil society and political will to tackle the problem, which those combating the fake Chinchona bark and quinine scandals in the 17^th^-19^th ^centuries would have found puzzling [10]. Importantly, African heads of state and President Chirac issued the Cotonou Appeal for more action against counterfeit medicines in Africa [49].

With artemisinin resistance in Asia, authorities there have a duty to contain resistant parasites so that they do not spread to Africa. African countries may wish to lobby for more political will in Asia for containment and for financial and human capacity support of African MRAs. The International Health Regulations (IHR)[50] may be a method of facilitating change. In the IHR "disease" means an "illness or medical condition, irrespective of origin or source, that presents or could present significant harm to humans". Poor quality medicines and inappropriate monotherapies-being man-made public health hazards-fall within this definition and the IHR could be invoked to try to stop the spread of poor quality medicines. In addition, a treaty, drafted under the auspices of the WHO, to bring international agreement on interventions to reduce the frequency of both substandard and counterfeit medicines would allow coordinated action [51]. Action is needed immediately or the hopes of controlling malaria in Africa may, again, be dashed.

## Conclusions

The description of a wide diversity of different counterfeit and substandard anti-malarials from eight sub-Saharan African countries are cause of great concern. Criminals are producing diverse harmful anti-malarial counterfeits with important public health consequences. The presence of artesunate monotherapy, substandard and/or degraded and counterfeit medicines containing sub-therapeutic amounts of unexpected anti-malarials will engender drug resistance. With the threatening spread of artemisinin resistance to Africa, much greater investment is required to ensure the quality of ACT and removal of artemisinin monotherapies. Support for MRAs is likely to be a key intervention. The International Health Regulations may need to be invoked to counter these poor quality medicines.

## Competing interests

The authors declare that they have no competing interests except CF who owns a consultancy company, East West Pharmaceuticals, which assist Chinese companies in clinical pharmaceutical development.

## Authors' contributions

AP, AET, AAA, SKO, SB, CF, JT, PF, HK, BA collected and documented samples. MDG, DCM, HN, LN, DMH, IS, GAH, KP, CWRS, KF, FMF performed the chemical/botanical analysis. PN performed packaging analysis. PN and KS performed the statistical analysis. PN, MDG, DCM, AET, PJG, FMF wrote the first draft. All authors revised and approved the final manuscript

## Supplementary Material

Additional file 1 **Reports of anti-malarial medicine quality in Africa**. Updated from Newton *et al *(2006a) and Amin & Kokwaro (2007). Data on medicine stability are not included. If chemical analysis detected API outside reference range, with no wrong API detected, but packaging was not analysed the sample was regarded as poor quality (PQ)-and could represent counterfeit (F), substandard (S) or degraded (D). C = convenience sample, r = convenience sample with some randomization, R = random sample, CR = case report, S = seizure, WHO = World Health Organization, USP = United States Pharmacopeia. Additional reports kindly provided by Roger Bate. Click here for file

Additional file 2 **Summary of packaging, chemical and botanical analysis of anti-malarial samples**. Results for tablets with AI chemical content < 90% or > 110% relative to the stated dose in red. Click here for file

Additional file 3 **Features of counterfeit artesunate labelled as made by Mekophar Chemical Pharmaceutical Joint-Stock Company**. Distinguishing features in red font. Click here for file

Additional file 4 **Distinguishing features of counterfeit DHA labelled as made by Jiaxing Nanhu Pharmaceutical Co**. Distinguishing features in red font. Click here for file

Additional file 5 **Distinguishing features of counterfeit DHA-piperaquine labelled as made by Zheijiang Holley Nanhu Pharmaceutical Co., Ltd. Distinguishing features in red font**. * medians, + medians (range). Click here for file

Additional file 6 **Distinguishing features of counterfeit artemether-lumefantrine labelled as manufactured by Beijing Novartis Pharma Ltd, Beijing, China for Novartis Pharma AG, Basle, Switzerland**. Distinguishing features in red font. Click here for file
