# Supplementary material for: Poor quality vital anti-malarials in Africa - an urgent neglected public health priority
Source: Malar J. 2011 Dec 13;10:352. doi: 10.1186/1475-2875-10-352 (PMC3262771; doi:10.1186/1475-2875-10-352)
Supplement: Additional file 1 — Reports of anti-malarial medicine quality in Africa. Updated from Newton et al (2006a) and Amin & Kokwaro (2007). Data on medicine stability are not included. If chemical analysis detected API outside reference range, with no wrong API detected, but packaging was not analysed the sample was regarded as poor quality (PQ)-and could represent counterfeit (F), substandard (S) or degraded (D). C = convenience sample, r = convenience sample with some randomization, R = random sample, CR = case report, S = seizure, WHO = World Health Organization, USP = United States Pharmacopeia. Additional reports kindly provided by Roger Bate. [file 1475-2875-10-352-S1.PDF]

Additional File 1. Reports of antimalarial medicine quality in Africa – updated from Newton *et al.* (2006a), Amin & Kokwaro (2007). Data on medicine stability are not included. If chemical analysis detected API outside reference range, with no wrong API detected, but packaging was not analysed the sample was regarded as poor quality (PQ) – and could represent counterfeit (F), substandard (S) or degraded (D). C= convenience sample, r = convenience sample with some randomisation, R = random sample, CR = case report, S = seizure, WHO = World Health Organization, USP = United States Pharmacopeia. Additional reports kindly provided by Roger Bate.

**Medicine**	**Country**	**Class**	**Trade Name**	**Stated Manufacturer**	**Formulation**	**Sampling method**	**Notes**	**Reference**
**Non-Artemisinins**								
Chloroquine	Angola	PQ			tab	CR	2/2 (100%) failed dissolution assays	Gaudiano *et al.* (2007)
Chloroquine	Burkina Faso	PQ			tab	C	24/39 (61%) samples failed chemical assays	Tipke *et al.* (2008)
Chloroquine	Cameroon	F, PQ			tab	C	6/32 (19%) failed assays, 1/32 (3%) 'substitution' & 1/32 (3%) défauts de fabrication'	WHO (1995)
Chloroquine sulphate 136 mg	Cameroon	F	'Nivaquine'		tab	CR	In tampered bottles, contained 2.5-18mg chloroquine phosphate/tablet	Basco *et al.* (1997)
Chloroquine	Cameroon	F, PQ			tab	C	6/11 (55%) of patients had taken chloroquine with no API detected, 1/11 (9%) had taken PQ chloroquine	Basco (2004)
Chloroquine	Cameroon	F, PQ			tab	C	42/133 (32%) counterfeit, 8/133 (6%) PQ	Basco (2004)
Chloroquine	Chad	GQ			tab	C	3/3 passed all tests	WHO (1995)

Chloroquine	Cote d'Ivoire	F	'Nirupquin'	'Syncom Formulations (India)'	tab	C	113.2% of stated API in 1/4 samples. 3/4 contained correct chloroquine content. A copy of Nivaquine of Aventis	Legris (2005)
Chloroquine	Gabon	GQ			syrup	C	0/8 failed chemical assays	WHO (2003)
Chloroquine	Gabon	PQ			tab	C	5/17 (29%) failed content assays & 1/7 (14%) failed dissolution tests	WHO (2003)
Chloroquine	Ghana	PQ			syrup	C	1/20 (5%) failed content assays	WHO (2003)
Chloroquine	Ghana	PQ			tab	C	12/18 (66.7%) failed content assays and 3/15 (20%) failed dissolution assays	WHO (2003)
Chloroquine	Guinea	F			oral	CR	Made of aspirin	WHO (1995)
Chloroquine	Guinea	F	'Nivaquine'	'Rhône Poulenc'	syrup	CR	No API detected, syrup more viscous than the genuine product	Barbereau (2006)
Chloroquine	Kenya	GQ			tab	C	All 13 samples passed assays	Kibwage *et al.* (1999)
Chloroquine	Kenya	PQ			tab & injection	C	4/29 (14%) tablet samples failed assays. The one injection sample passed	Thoithi et al. (2002)
Chloroquine	Kenya	PQ			Syrup	C	2/8 (25%) failed content assays	WHO (2003)
Chloroquine	Kenya	PQ			tab	C	3/7 (42.8%) failed content assays & 2/7 (28.6%) failed dissolution assays	WHO (2003)

Chloroquine	Madagascar	S			tab	C	2/8 (25%) défauts de fabrication'	WHO (1995)
Chloroquine	Mali	PQ			syrup	C	4/6 (66.7%) failed content assays	WHO (2003)
Chloroquine	Mali	PQ			tab	C	9/19 (47.3%) failed content assays and 1/19 (5.2%) failed dissolution tests	WHO (2003)
Chloroquine	Mozambique	PQ			Syrup	C	3/12 (25%) failed content assays	WHO (2003)
Chloroquine	Mozambique	PQ			tab	C	3/15 (20%) failed content assays & 1/15 (6.7%) failed dissolution-tests	WHO (2003)
Chloroquine	Nigeria	F, PQ			oral	C	2/32 (8%) no API detected, 8/32 failed BP assays but chloroquine detected	Shakoor *et al.* (1997)
Chloroquine phosphate	Nigeria	PQ			Caps Syrups Tab Injection	R	9/20 (70%), 20/20 (100%), 17/18 (94%), 14/15 (93%) outside British Pharmacopeia limits	Taylor *et al.* (2001)
Chloroquine sulphate	Nigeria	PQ			Syrups Tab Capsules	R	8/11 (73%), 15/19 (93%), 0/1 outside British Pharmacopeia limits	Taylor *et al.* (2001)
Chloroquine	Nigeria	F				CR	Chlorpheniramine sold as chloroquine	Erhun *et al.* (2001)
Chloroquine	Nigeria	F	'Nivaquine'		tab	S	Fake labels seized	Edike (2003)
Chloroquine	Nigeria	F	'Nivaquine'		syrup	S	Fake labels seized	Edike (2003)
Chloroquine	Abeokuta, Nigeria	F, S				C	3/50 (6%) no API detected, 16/50 (32%) substandard	Idowu *et al.* (2006)

Chloroquine	Nigeria	PQ			tab, syrup and injection	C	'Over 85% of (14) tablet samples complied' with content assays and 21% failed friability test. Over 90% of (13) syrups failed content analysis (API too high) and 23% failed microbial growth test. All 5 injections failed content analysis. All tablets passed dissolution/disintegrati on tests	Aina *et al.* (2007)
Chloroquine	Nigeria	F, PQ			tab	C	2/56 failed chemical assays, 1/56 no API detected	Onwujekwe *et al.* (2009)
Chloroquine	Senegal	PQ			tab	C	35% failed USP monograph tests	Smine *et al.* (2002)
Chloroquine phosphate 250mg	Sierra Leone	F		'The Wallis Laboratory, 11 Camford Rd., Luton' UK	tab	CR	Made of aspirin	Sesay (1988)
Chloroquine	Sudan	S			tab	Bio	1/5 had significantly lower bioavailability than the other 4/5	Mahmoud *et al.* (1994)
Chloroquine	Sudan	PQ			tab	CR	8% tablets failed chemical % API analysis and 8% failed dissolution tests	Alfadl *et al.* (2006)
Chloroquine	Sudan	PQ			syrup	CR	Some syrups and suspension failed visual inspection	Alfadl *et al.* (2006)
Chloroquine	Sudan	GQ			injection	CR	All passed visual inspection and content analysis	Alfadl *et al.* (2006)

Chloroquine phosphate 250mg and chloroquine sulphate 100mg	Tanzania	PQ			tab	C	1/3 failed content assay at 92% API. All passed dissolution.	Anthony & Temu-Justin (1999)
Chloroquine phosphate 150 mg base	Tanzania	GQ	‘Dawaquine’ and ‘Kabi-Vitrum’ preparation	‘DAWA, Dar es Salaam, Tanzania and Kabi-Vitrum, Stockholm’	tab	Bio	Passed bioequivalence testing	Nsimba *et al.* (2001)
Chloroquine phosphate 250mg	Tanzania	GQ	7 brands		tab	C	Passed content and dissolution-tests	Risha *et al.* (2002)
Chloroquine	Tanzania	S	Sugar coated ‘Dawaquin’	‘Dawa Pharmaceuticals, Nairobi’	tab	CR	99% API but low bioavailability	Rimoy *et al.* (2002)
Chloroquine	Tanzania	PQ	9 brands		tab	C	All 9 passed content assay but 1/9 failed dissolution testing. The failed sample was sugar coated	Abdi et al. (1995)
Chloroquine	Uganda	PQ			tab and injection	C	Tablets 22/40 (55%) and injection 30/48 (63%) failed assay for chloroquine content	Ogwal-Okeng *et al.* (1998)
Chloroquine	Uganda	PQ			tab and injection	C	Tablets 18/47 (39%) and injection 23/45 (51%) failed assay for chloroquine content	Ogwal-Okeng *et al.* (2003)
Chloroquine	Zimbabwe	PQ			syrup	C	2/15 (13.3%) failed content assays	WHO (2003)
Chloroquine	Zimbabwe	PQ			tab	C	8/14 (57.1%) failed content assays and 1/14 (7.1%) failed dissolution tests	WHO (2003)

Amodiaquine	Burkina Faso	GQ			tab	C	0/6 samples failed chemical assays	Tipke *et al.* (2008)
Amodiaquine	Ghana	PQ			tab	C	2/6 (33%) failed TLC and/or dissolution tests	Bate *et al.* (2008)
Amodiaquine	Kenya	GQ			tab	C	One sample of one passed assays	Kibwage *et al.* (1999)
Amodiaquine	Kenya	GQ			tab and suspension	C	One sample each of tablets and suspension passed	Thoithi *et al.* (2002)
Amodiaquine	Kenya	PQ			tab<br>syrup	R	11/29 (38%) tablet samples & 7/23 (30%) syrups failed content and/or dissolution tests	Amin *et al.* (2005)
Amodiaquine	Kenya	PQ			tab	C	4/8 (50%) failed TLC and/or dissolution tests	Bate *et al.* (2008)
Amodiaquine	Nigeria	PQ			tab	C	1/4 (25%) failed TLC and/or dissolution tests	Bate *et al.* (2008)
Amodiaquine	Senegal	GQ			tab	C	'all of the amodiaquine samples passed the monograph tests'	Smine *et al.* (2002)
Amodiaquine	Tanzania	S	'Emoquin'	'Made in Kenya'	tab	C	2 (both 'Emoquin')/15 (13%) failed dissolution tests	Minzi *et al.* (2003)
Amodiaquine	Tanzania	PQ			tab	C	6/100 failed chemical tests	Kaur *et al.* (2008)
Amodiaquine	Tanzania	PQ			tab	C	2/2 (100%) failed TLC and/or dissolution tests	Bate *et al.* (2008)
Amodiaquine	Uganda	PQ			tab	C	5/9 (56%) failed TLC and/or dissolution tests	Bate *et al.* (2008)
Amodiaquine	Nigeria	PQ			tabs	R	1/5 failed dissolution tests	Odunfa *et al.* (2009)

Primaquine phosphate	Namibia	PQ			tab	CR	Peace Corp volunteer contracted *P. vivax* during prophylaxis with primaquine containing 12mg/tablet, as opposed to genuine 26.3mg	Kron (1996)
Halofantrine	Ghana, Nigeria, Sierra Leone	F	'Halfan'	'GSK'	syrup	CR	No API detected, contained sulfamethazine or sulfisomidine	Wolff *et al.* (2003), Anon (2003a), Cockburn *et al.* (2005)
Halofantrine	Nigeria	F	'Halfan'		tab	CR	Fake labels seized	Edike (2003)
Halofantrine	Nigeria	F	'Halfan'		tab	CR	Smuggled amongst imported clothes and boots.	Oyeniran (2007)
Halofantrine	Tanzania	F	'Halfan'		tab	CR	2005, Counterfeiters extended shelf life of expired halofantrine by 2 years	Ndomondo-Sigonda (2005)
Quinine	Angola	PQ			tab	CR	1/2 failed dissolution tests	Gaudiano *et al.* (2007)
Quinine	Burkina Faso	PQ			tab	C	3/9 samples failed chemical assays	Tipke *et al.* (2008)
Quinine	Burundi	F			tab	CR	1/3 (30%) failed content assay. Contained chloroquine	Gaudiano *et al.* (2007)
Quinine	Cameroon	F			tab and injection	C	1/24 (4%) no quinine detected (tablet)	WHO (1995)
Quinine	Cameroon	F			tab	C	2/4 (50%) of patients who had taken 'quinine' had taken fakes containing chloroquine	Basco (2004)

Quinine	Cameroon	F, PQ			tab	C	7/70 (10%) contained no API; 45/70 (64%) probably PQ	Basco (2004)
Quinine	Chad	GQ			tab and injection	C	6/6 passed all tests	WHO (1995)
Quinine 300mg	DR Congo	PQ			tab	CR	88.6% quinine present	Gaudiano *et al.* (2006)
Quinine	DR Congo	PQ			tab	CR	1/10 (10%) failed uniformity of mass assay, 3/10 (30%) failed dissolution tests and 1/10 (10%) failed content assay. Total failures were 4/10 (40%)	Gaudiano *et al.* (2007)
Quinine sulphate	Ghana	F			?	S	Stated to be counterfeit	Ghana News Agency (2010)
Quinine	Kenya	PQ			Raw material, tabs, syrup & injection	C	½ raw material failed, 2/2 tablets, 1/1 syrups and 1/1 injections passed	Thoithi *et al.* (2002)
Quinine	Madagascar	S			tab or injection	C	1/11 (9%) 'défauts de fabrication'	WHO (1995)
Quinine sulphate	Nigeria	F			tab	Bio	sugar-coated tablets with no quinine detected. Two other oral preparations passed bioequivalence testing	Sowunmi *et al.* (1994)
Quinine hydrochloride	Nigeria	GQ			injection	R	10/10 passed British Pharmacopeia limits	Taylor *et al.* (2001)

Quinine sulphate	Nigeria	PQ			syrup<br>tab	R	1/1 & 4/17 (24%) failed British Pharmacopeia limits	Taylor *et al.* (2001)
Quinine	Nigeria	F			tab	R	13/28 (46%) failed chemical assays, 7/28 (25%) contained chloroquine	Onwujekwe *et al.* (2009)
Quinine	Rwanda	F				CR	Fake 'quinine is in the form of yellow-coated tablets in a white cylindrical plastic tin with the tablets being housed in double layer transparent cellophane sachets'	Anon (2001)
Quinine	Sudan	PQ			injection	CR	Some failed due to change in colour	Alfadl *et al.* (2006)
Quinine	Sudan	GQ			tab	CR	All samples (≥50) passed assays	Alfadl *et al.* (2006)
Quinine	Tanzania	F		'company in Cyprus'	injection	CR	2001, expired chloroquine injection (from an unregistered Indian company) was relabeled as Quinine Dihydrochloride Injection 600mg/2ml from a company in Cyprus	Ndomondo-Sigonda (2005)
Quinine	Tanzania	F			tab	CR	2005, No API detected	Ndomondo-Sigonda (2005)
Quinine	Tanzania	PQ			tab	R	15/63 failed chemical tests	Kaur *et al.* (2008)

Quinine salts	Tanzania	PQ				C	2/81 (2%) failed tests at Quality Control Laboratory during one year	Risha *et al.* (2008)
Quinine	Uganda	F		'Kampala Pharmaceutical Industries'	tab	CR	Counterfeit packaging, no API detected	Bogere & Nafula (2007)
Quinine	Uganda	F			tab	CR	Tin of 'magnesium' relabelled as quinine	Mugabe (2009)
Quinine	Uganda	F			tab	CR	Chloroquine relabelled as quinine	Mugabe (2009)
Mefloquine	Angola	GQ			tab	CR	2/2 passed API content assays	Gaudiano *et al.* (2007)
Mefloquine	Ghana	GQ			tab	C	0/1 failed TLC and/or dissolution tests	Bate *et al.* (2008)
Mefloquine	Nigeria	PQ				C	1/2 (50%) failed TLC and/or dissolution	Bate *et al.* (2008)
Mefloquine	Senegal	? PQ			tab	CR	Failed prophylaxis. Tablets not available for analysis	Reidenberg *et al.* (2001)
Mefloquine	Sudan	GQ			tab	CR	Passed assays	Alfadl *et al.* (2006)
Mefloquine	Tanzania	GQ				C	0/3 failed TLC and/or dissolution tests	Bate *et al.* (2008)
Mefloquine	Uganda	PQ				C	3/11 (27%) failed TLC and/or dissolution tests	Bate *et al.* (2008)
Sulphadoxine-pyrimethamine	Burkina Faso	F			tab	C	1/9 samples failed chemical assays. Probably contained metronidazole	Tipke *et al.* (2008)
Sulphadoxine-pyrimethamine	Cameroon	F			oral	C	1/9 (11%) no API detected	WHO (1995)
Sulphadoxine-pyrimethamine	Cameroon	F			tab		10/78 (13%) no API detected	Basco (2004)

Sulphadoxine-pyrimethamine	Cameroon	PQ		'Ajanta Pharma Limited, Mauritius'	tab	r	Uniformity of mass non-compliant	WHO (2011)
Sulphadoxine-pyrimethamine	Cameroon	PQ		'Britlodge Ltd, UK'	tab	r	Sulphadoxine-pyrimethamine failed dissolution tests	WHO (2011)
Sulphadoxine-pyrimethamine	Cameroon	PQ		'Simrone Pharamceuticals Industries Ltd, India'	tab	r	Tablets chipped. Pyrimethamine 87.2% API. Pyrimethamine failed dissolution tests	WHO (2011)
Sulphadoxine-pyrimethamine	Cameroon	PQ		'Swiss Pharma Nigeria Ltd, Nigeria'	tab	r	Pyrimethamine failed dissolution tests	WHO (2011)
Sulphadoxine-pyrimethamine	Congo	PQ			tab	CR	6/10 (60%) failed dissolution tests	Gaudiano *et al.* (2007)
Sulphadoxine-pyrimethamine	Chad	GQ			oral	C	3/3 passed % API HPLC assays	WHO (1995)
Sulphadoxine-pyrimethamine	Côte d'Ivoire	F	'Maloxine'	'Encore Pharmaceuticals India'	tab	C	Contains 102.5% stated dose of sulphadoxine and 85.9% stated dose of pyrimethamine. Copy of packaging and false information on origin given	Legris (2005)
Sulphadoxine-pyrimethamine	Côte d'Ivoire	F		'Brown & Burk Pharmaceutical Ltd. India	tab	C	Copy of packaging and false information on origin given. Contains correct amount of API	Legris (2005)
Sulphadoxine-pyrimethamine	Côte d'Ivoire	F		'Sarvodaya Laboratories, India	tab	C	Copy of packaging and false information on origin given. Contains correct amount of API	Legris (2005)
Sulphadoxine-pyrimethamine	Côte d'Ivoire	F		'India'	tab	C	Copy of packaging and false information on origin given. Contains correct amount of API	Legris (2005)

Sulphadoxine-pyrimethamine	Côte d'Ivoire	F	'Melaxime'	'Syncom Formulations, India'	tab	C	Copy of packaging. Contains correct amount of API	Legris (2005)
Sulphadoxine-pyrimethamine	Gabon	GQ			tab	C	0/10 failed content assays	WHO (2003)
Sulphadoxine-pyrimethamine	Ghana	PQ			tab	C	3/8 (37.5%) failed content assays & ¾ (75%) failed dissolution tests	WHO (2003)
Sulphadoxine-pyrimethamine	Ghana	PQ			tab	C	3/6 (50%) failed TLC and/or dissolution tests	Bate *et al.* (2008)
Sulphadoxine-pyrimethamine	Ghana	PQ		'Ally Pharma Options, India'	tab	r	Pyrimethamine failed dissolution tests. Contaminant on TLC	WHO (2011)
Sulphadoxine-pyrimethamine	Ghana	PQ		'Kinapharma Ltd, Ghana'	tab	r	Pyrimethamine 83.9% API. Pyrimethamine failed dissolution tests	WHO (2011)
Sulphadoxine-pyrimethamine	Ghana	PQ		'Mission Pharmaceuticals Ltd, India'	tab	r	Uniformity of mass non-compliant	WHO (2011)
Sulphadoxine-pyrimethamine	Ghana	PQ		'Phyto-Riker Pharmaceuticals Ltd, Ghana'	tab	r	Sulphadoxine and pyrimethamine failed dissolution tests	WHO (2011)
Sulphadoxine-pyrimethamine	Ghana	PQ		'Uni-Med, India'	tab	r	Pyrimethamine failed dissolution tests	WHO (2011)
Sulphadoxine-pyrimethamine	Kenya	GQ	'Fansidar' 'Falcidin'	'Hoffman-La Roche, Basel, Switzerland' and 'Cosmos Industries, Nairobi, Kenya'	tab	Bio	No significant difference in bioavailability	Murphy & Mberu (1994)
Sulphadoxine-pyrimethamine	Kenya	GQ			tab	C	All 3 samples passed assays	Kibwage *et al.* (1999)

Sulphadoxine-pyrimethamine	Kenya	PQ			tab	C	1/17 (6%) brands had 84.3% sulphadoxine and 83.3% pyrimethamine. 23/33 (70%) samples failed dissolution tests	Kibwage & Ngugi (2000)
Sulphadoxine-pyrimethamine	Kenya	PQ			tab & suspension	C	15/39 (38%) tablet samples failed and 2/3 (67%) suspensions failed content assays	Thoithi *et al.* (2002)
Sulphadoxine-pyrimethamine	Kenya	PQ			tab	C	0/12 failed content assays & 11/12 (91.7%) failed dissolution tests	WHO (2003)
Sulphadoxine-pyrimethamine	Kenya	PQ			tab<br>syrup	R	13/37 (35%) tablet samples & 16/27 (59%) syrup failed content and/or dissolution test	Amin *et al.* (2005)
Sulphadoxine-pyrimethamine	Kenya	PQ			tab	C	6/16 (38%) failed TLC and/or dissolution tests	Bate *et al.* (2008)
Sulphadoxine-pyrimethamine	Madagascar	GQ			tab	C	5/5 passed all tests	WHO (1995)
Sulphadoxine-pyrimethamine	Madagascar	PQ	‘Fastidar’, ‘Medodar’, Paludamine’ , ‘Paludoxine’		tab	r	11/21 (52%) failed dissolution tests	USP (2009a)
Sulphadoxine-pyrimethamine	Mali	PQ			tab	C	0/7 failed content assays & 7/7 (100%) failed dissolution tests	WHO (2003)
Sulphadoxine-pyrimethamine	Mozambique	PQ			tab	C	1/18 (5.5%) failed content assays & 18/18 (100%) failed dissolution tests	WHO (2003)

Sulphadoxine-pyrimethamine	Nigeria	F	'Fansidar'	'Roche'	tab	CR	No sulphadoxine or pyrimethamine detected. Contained 5mg chloramphenicol/tablet	ten Ham (1992)
Sulphadoxine-pyrimethamine	Nigeria	PQ			tab<br>syrup	R	2/50 (4%) tabs failed pyrimethamine, 11/50 tabs failed sulphadoxine. 3/13 (23%) syrups failed pyrimethamine and 5/13 (38%) failed sulphadoxine by British Pharmacopeia for API % content	Taylor *et al.* (2001)
Sulphadoxine-pyrimethamine	Nigeria	S			tab	C	5/8 (63%) brands (all labelled as made in India) failed friability tests, dissolution tests or API % content assays	Odeniyi *et al.* (2003)
Sulphadoxine-pyrimethamine	Nigeria	F	'Fansidar'		tab	CR	Paracetamol tablets substituted for SP	Anon (2003b)
Sulphadoxine-pyrimethamine	Nigeria	F	'Fansidar'<br>'Maloxine'		tab	CR	Seizure of fake labels	Edike (2003)
Sulphadoxine-pyrimethamine	Nigeria	F		'Roche'	tab	CR	No API detected, contained chloramphenicol 5mg	Deisingh (2005)
Sulphadoxine-pyrimethamine	Nigeria	PQ			tab	C	1/2 (50%) failed TLC and/or dissolution tests	Bate *et al.* (2008)

Sulphadoxine-pyrimethamine	Nigeria	F	'Maloxine'			CR	348,000 tablets in sachets of three tablets each, contained in 6,960 boxes packed in 960 cartons with Batch No: EM-396 and manufacturing and expiring dates 04/2008 and 03/2011. In container, shipped from China, claimed to contain sellotape	Ogundipe (2009)
Sulphadoxine-pyrimethamine	Nigeria	F	'Amalar'		tab	CR	294,000 sachets of 'Amalar' packed as three tablets each, contained in 5,880 boxes packed in 196 cartons with Batch No: ARTP 0053 and manufacturing and expiring dates of January 2007 and January 2010 respectively. In container, shipped from China, claimed to be sellotape	Ogundipe (2009)
Sulphadoxine-pyrimethamine	Nigeria	PQ			tab	R	44/113 (39%) failed API% HPLC assays and/or dissolution tests	Onwujekwe *et al.* (2009)

Sulphadoxine-pyrimethamine	Nigeria	PQ		'Bond Chemical Ind. Ltd, Nigeria'	tab	r	Pyrimethamine 88.6% API. Pyrimethamine failed dissolution tests	WHO (2011)
Sulphadoxine-pyrimethamine	Nigeria	PQ		'Emzor Pharm Ind. Ltd, Nigeria'	tab	r	4/4 (100%) pyrimethamine failed dissolution tests. 1/4 (25%) sulphadoxine failed dissolution tests, 2/4 (50%) uniformity of mass non-compliant	WHO (2011)
Sulphadoxine-pyrimethamine	Nigeria	PQ		'Evans Medical Plc, Nigeria'	tab	r	Sulphadoxine & pyrimethamine failed dissolution. Uniformity of mass non-compliant	WHO (2011)
Sulphadoxine-pyrimethamine	Nigeria	PQ		'May & Baker Nigeria Plc, Nigeria'	tab	r	Sulphadoxine & pyrimethamine failed dissolution tests	WHO (2011)
Sulphadoxine-pyrimethamine	Nigeria	PQ		'Medrel Pharmaceuticals Ltd, India'	tab	r	Uniformity of mass non-compliant	WHO (2011)
Sulphadoxine-pyrimethamine	Nigeria	PQ		'Neimeth International Pharmaceuticals Plc, Nigeria'	tab	r	Uniformity of mass non-compliant	WHO (2011)

Sulphadoxine-pyrimethamine	Nigeria	C		'Shreechem Lab, India'	tab	r	Sulphadoxine 9.1% API. Pyrimethamine not detected. Sulphadoxine failed dissolution tests. Tablets mottled. Tablets differed from other batches of same 'product'	WHO (2011)
Sulphadoxine-pyrimethamine	Nigeria	PQ		'Shreechem Lab, India'	tab	r	Pyrimethamine failed dissolution tests	WHO (2011)
Sulphadoxine-pyrimethamine	Nigeria	PQ		'Swiss Pharma Nigeria Ltd, Nigeria'	tab	r	Sulphadoxine & pyrimethamine failed dissolution tests	WHO (2011)
Sulphadoxine-pyrimethamine	Nigeria	PQ		'VITAPHOS Laboratory Nigeria Ltd, Nigeria'	tab	r	Pyrimethamine 80.2% API. Sulphadoxine & pyrimethamine failed dissolution tests	WHO (2011)
Sulphadoxine-pyrimethamine	Rwanda	S		'Labophar, Butare, Rwanda'	tab	C	Failed pyrimethamine release requirement on dissolution testing	Kayumba *et al.* (2004)
Sulphadoxine-pyrimethamine	Rwanda	PQ			tab	C	3/6 (50%) failed TLC and/or dissolution tests	Bate *et al.* (2008)
Sulphadoxine-pyrimethamine	Senegal	PQ			tab	C	55% failed monograph tests	Smine *et al.* (2002)
Sulphadoxine-pyrimethamine	Senegal	PQ	'Doximine', 'Madar', 'Malastop', 'Maloxine', 'Melaxime'		tab	r	13/27 failed dissolution tests	USP (2009a)
Sulphadoxine-pyrimethamine	Sierra Leone	F		'Roche'	tab	CR	Counterfeit	Sesay (1988)
Sulphadoxine-pyrimethamine	Sudan	PQ			tab	C	0/20 failed content assays & 12/15 (80%) failed dissolution tests	WHO (2003)

Sulphadoxine-pyrimethamine	Tanzania	S			tab	C	All 9 brands passed content assay. 4/9 (44%) brands failed dissolution assays	Jande *et al.* (2000)
Sulphadoxine-pyrimethamine	Tanzania	S	'Flamingo Pharmaceuticals', 'Shelys Pharmaceutical Industries'	'India' 'Tanzania'	tab	C	2/4 (50%) did not meet tolerance limits for dissolution tests	Risha *et al.* (2002)
Sulphadoxine-pyrimethamine	Tanzania	S	'Falsitat' 'Malaridox' 'SP' Sulphadar'	'Cyprus India Tanzania Tanzania'	tab	C	2/18 (11%) & 8/18 (44%) samples failed the assay for content and dissolution tests, respectively	Minzi *et al.* (2003)
Sulphadoxine-pyrimethamine	Tanzania	S	'Tansidar' 'Malostat' 'Orodar' 'Malareich'	'Tansidar (Tanzania), Malostat (India), Orodar (Kenya), Malareich (India)'	tab	C	4/11 (36%) brands failed hardness, friability or disintegration tests. All contained an impurity, closely related to sulphadoxine at ~ 0.5% w⁄w.	Hebron *et al.* (2005)
Sulphadoxine-pyrimethamine	Tanzania	S	'Amaximum'	'Tanzania'	tab	Bio	Inferior bioavailability in comparison to Fansidar	Minzi *et al.* (2006)
Sulphadoxine-pyrimethamine	Tanzania	PQ			tab	R	8/58 failed chemical tests	Kaur *et al.* (2008)
Sulphadoxine-pyrimethamine	Tanzania	PQ			tab	C	3/11 (27%) failed TLC and/or dissolution tests	Bate *et al.* (2008)

Sulphadoxine-pyrimethamine	Tanzania	PQ			tab	C	39/258 (15%) failed tests at Quality Control Laboratory during one year	Risha *et al.* (2008)
Sulphadoxine-pyrimethamine	Tanzania	PQ		'Shely's Pharmaceuticals Ltd, Tanzania'	tab	r	Pyrimethamine failed dissolution tests	WHO (2011)
Sulphadoxine-pyrimethamine	Tanzania	PQ		'Shely's Pharmaceuticals Ltd, Tanzania'	tab	r	Uniformity of mass non-compliant	WHO (2011)
Sulphadoxine-pyrimethamine	To Togo via Belgium	F	'Fansidar'	'Roche'	tab	CR	In transit from Mumbai, India, via Morocco	Anon (2008)
Sulphadoxine-pyrimethamine	Uganda	PQ			tab	C	3/9 (33%) failed TLC and/or dissolution tests	Bate *et al.* (2008)
Sulphadoxine-pyrimethamine	Uganda	PQ	'Agosidar', 'L-Kelfin', 'Malagon'		tab	r	7/43 (16%) failed dissolution tests and 3/43 (7%) failed content assay	USP (2009a)
Sulphadoxine-pyrimethamine	Zambia	F				CR	Contains paracetamol	Koch *et al.* (1994)
Sulphadoxine-pyrimethamine	Zimbabwe	PQ			tab	C	1/10 (10%) failed content assays & 10/10 (100%) failed dissolution tests	WHO (2003)
Sulfalene-pyrimethamine	Ghana, Nigeria	F	'Metakelfin'	'Pharmacia'	oral	CR	White sachets withdrawn and replaced by packaging of silver foil. Fakes contained paracetamol	Ghana News Agency (2002)

Sulfalene-pyrimethamine	Ghana	F	'Metakelfin'	'Pharmacia & Upjohn'	oral	S	Counterfeit packaging	Ghana News Agency (2010)
Sulfalene-pyrimethamine	Kenya	PQ			tab & 'drops'	C	1/1 tablet sample passed and 1/2 'drops' failed content assay	Thoithi *et al.* (2002)
Sulfalene-pyrimethamine	Kenya	F	'Metakelfin'		tab		No API, contained paracetamol	Jähnke (2004)
Sulfalene-pyrimethamine	Nigeria	PQ		'Drugfield Pharmaceuticals Ltd, Nigeria'	tab	r	1/1 pyrimethamine failed dissolution tests	WHO (2011)
Sulfalene-pyrimethamine	Tanzania	F	'Metakelfin'	'Pharmacia and Upjohn' 'Farmitalia'	tab	CR	Contained paracetamol	Ndomondo-Sigonda (2005)
Sulfalene-pyrimethamine	Tanzania	F	'Metakelfin'			CR	Notified by good Samaritan. Batch E378A (10/2006-10/2010) no pyrimethamine detected, 0.4% sulfalene. Batch E894A (10/2008-01/2012) detected, 75.6% sulfalene. Fake blister shining (rather than 'dim') silver with instructions in English & French (rather than only English) with embossed codes. Five arrested.	Shekighenda (2009)

Sulfalene-pyrimethamine	Tanzania	PQ			tab	R	9/42 failed API % HPLC assays and dissolution tests	Kaur *et al.* (2008)
Sulfalene-pyrimethamine	Tanzania	F	'Metakelfin'		tab	CR	Fakes found in 40 pharmacies	Economist Intelligence Unit (2009)
Sulfalene-pyrimethamine	Tanzania	PQ		'Shely's Pharmaceuticals Ltd, Tanzania'	tab	r	Pyrimethamine failed dissolution tests	WHO (2011)
Sulfalene-pyrimethamine	Uganda	F	'Metakelfin'		tab	CR	fake	Mugabe (2009)
Tetracycline	Cameroon	GQ			tab	C	13/13 passed HPLC API% assays and mass uniformity tests	WHO (1995)
Tetracycline	Chad	GQ			tab	C	4/4 passed HPLC API% assays and mass uniformity tests	WHO (1995)
Tetracycline	Madagascar	GQ			tab	C	8/8 passed HPLC API% assays and mass uniformity tests	WHO (1995)
Tetracycline	Nigeria	PQ			caps	C	1/10 failed British Pharmacopeia HPLC API% assays	Shakoor *et al.* (1997)
Tetracycline	Nigeria	F				CR	Relabelled expired drugs	Erhun *et al.* (2001)
Doxycycline	Nigeria	PQ			caps	R	6/19 (68%) failed British Pharmacopeia HPLC API% assays	Taylor *et al.* (2001)

Proguanil	Nigeria	GQ			tab	R	19/19 passed British Pharmacopeia HPLC API% assays	Taylor *et al.* (2001)
Amodiaquine, mefloquine and sulphadoxine–pyrimethamine	Accra, Ghana	GQ, PQ			tab	r	2007 5/13 (38%), 2010 5/23 (22%) failed Minilab tests<br><br>2007 1/13 (8%), 2010 2/23 (9%) failed visual inspection	Bate & Hess (2010)
Amodiaquine, mefloquine and sulphadoxine–pyrimethamine	Lagos, Nigeria	GQ, PQ			tab	r	2007 3/8 (38%), 2008 8/59 (14%), 2010 5/30 (17%) failed Minilab tests<br><br>2007 1/8 (13%), 2008 3/59 (51%), 2010 4/30 (13%) failed visual inspection	Bate & Hess (2010)
**Artemisinin monotherapies and ACTs**								
Artesunate	Burkina Faso	PQ, (S or D ?)			tab	C	1/9 artesunate samples in poor physical condition	Tipke *et al.* (2008)
Artesunate	Cameroon	F	'Arsuman'	'Sanofi-synathelabo'	tab	CR	Copy of 'Arsumax'. Contained correct API% (50mg/tablet) by HPLC	Newton *et al.* (2006b)

Artesunate	Chad	F			tab	CR	Counterfeit	Aline Plançon pers. obs
Artesunate 100mg	DR Congo	F	'Saphnate'	'Saphire sprl' 'Belgium'	tab	C	Contained 98.9% API. Stated manufacturer 'Saphire sprl'' does not exist in Belgium at address stated	Atemnkeng *et al.* (2007)
Artesunate	Ghana	S			tab	C	14 of 17 (82.4%) sampled artesunate tablets sold in pharmacies in Kumasi failed to meet European Pharmacopeia content requirements	Ofori-Kwakye *et al*. (2008)
Artesunate	Ghana	PQ			tab	C	3/8 (38%) failed TLC and/or dissolution tests	Bate *et al.* (2008)
Artesunate	Ghana	F	'Artesunate'	'Guilin Pharmaceuticals Co. Ltd'	tab	CR	Counterfeit packaging	Ghana News Agency (2011)
Artesunate	Kenya	PQ	'Gsunate Forte'	'GVS'	tab	C	Contained 88.5% API	Atemnkeng *et al.* (2007)
Artesunate	Kenya	PQ			tab	C	0/0 failed TLC and/or dissolution tests	Bate *et al.* (2008)
Artesunate	Nigeria	F			tab	S	Smuggled in handbags	Oyeniran (2006)
Artesunate	Nigeria	F			tab	S	Smuggled amongst imported clothes and boots. Contained 60-65% API	Oyeniran (2007)
Artesunate	Nigeria	PQ			tab	Bio	1/9 (11%) brands failed dissolution tests	Esimone *et al.* (2008a)
Artesunate	Nigeria	S		Failed brands were labelled as from 'India, Vietnam, Nigeria and China'	tab	C	5/9 (56%) brands failed UV spectroscopic assays	Esimone *et al.* (2008b)

Artesunate	Nigeria	PQ			tab	C	2/6 (33%) failed TLC and/or dissolution tests	Bate *et al.* (2008)
Artesunate	Nigeria	GQ			tab	C	0/24 failed API% content HPLC assays or dissolution tests	Onwujekwe *et al.* (2009)
Artesunate	Nigeria	PQ			tab	R	2/15 contained <50% API	Odunfa *et al.* (2009)
Artesunate	Nigeria	F	'Artesunat'		tab	S	Imported from Hong Kong	Udoh (2010b)
Artesunate	Tanzania	PQ			tab	C	4/13 (31%) failed TLC and/or dissolution	Bate *et al.* (2008)
Artesunate	Tanzania	GQ				C	0/22 failed tests at Quality Control Laboratory during one year	Risha *et al.* (2008)
Artesunate	Uganda	PQ			tab	C	6/18 (33%) failed TLC and/or dissolution tests	Bate *et al.* (2008)
Artemether 50mg	DR Congo	F	'Artesaph'	'Saphire sprl' Belgium	tab	C	Contained 100.6% API. Stated manufacturer 'Saphire sprl'' does not exist in Belgium at address stated	Atemnkeng *et al.* (2007)
Artemether	Ghana	PQ			tab	C	0/3 failed TLC and/or dissolution tests	Bate *et al.* (2008)
Artemether 150mg	Kenya	PQ	'Emal'	'Themis'	Im	C	Contained 77% API	Atemnkeng *et al.* (2007)
Artemether	Kenya	PQ			tab	C	1/1 (100%) failed TLC and/or dissolution tests	Bate *et al.* (2008)
Artemether	Sudan	GQ			inj	CR	Passed assays	Alfadl *et al.* (2006)
Artemether	Uganda	PQ			tab	C	2/7 (29%) failed TLC and/or dissolution tests	Bate *et al.* (2008)

Dihydroartemisinin 60 mg	DR Congo	F	'Salaxin'	'Saphire sprl' Belgium	tab	C	Contained 87.7% API. Stated manufacturer 'Saphire sprl'' does not exist in Belgium at address stated	Atemnkeng *et al.* (2007)
Dihydroartemisinin	Ghana	PQ			tab	C	2/5 (40%) failed TLC and/or dissolution tests	Bate *et al.* (2008)
Dihydroartemisinin	Kenya	F	'Cotecxin'	'Beijing COTEC New Technology Corp'	tab	W	counterfeit	Kenya Pharmacy Board (2007)
Dihydroartemisinin 60 mg	Kenya	F	'Cotecxin'	'Beijing COTEC New Technology Corp'	tab	CR	No API detected	Newton *et al.* (2006b)
Dihydroartemisinin 160mg	Kenya	PQ	'Alaxin'	'GVS Labs'	Powder	C	Contained 81% API	Atemnkeng *et al.* (2007)
Dihydroartemisinin 160mg	Kenya	PQ	'Santecxin'	'Shsj, China'	Powder	C	Contained 78% API	Atemnkeng *et al.* (2007)
Dihydroartemisinin	Kenya	F	'Cotexin'		tab	S	Smuggled in handbags	Oyeniran (2006)
Dihydroartemisinin	Kenya	PQ			tab	C	5/9 (56%) failed TLC and/or dissolution tests	Bate *et al.* (2008)
Dihydroartemisinin	Nigeria	PQ			tab	C	1/1 (100%) failed TLC and/or dissolution tests	Bate *et al.* (2008)
Dihydroartemisinin	Nigeria	F			tab	CR	1/4 (25%) no API detected	Onwujekwe *et al.* (2009), Ioset & Kaur (2009)
Dihydroartemisinin	Tanzania	PQ			tab	C	2/4 (50%) failed TLC and/or dissolution tests	Bate *et al.* (2008)
Dihydroartemisinin	Tanzania	F	'Cotecxin'	'Beijing COTEC New Technology Corp'	tab	CR	No API detected	Arrow (2001)

Dihydroartemisinin	Uganda	PQ			tab	C	2/3 (67%) failed TLC and/or dissolution tests	Bate *et al.* (2008)
Artemisinin monotherapies	Accra, Ghana	GQ, PQ			tab	r	2007 5/16 (31%), 2010 5/23 (22%) failed Minilab tests<br><br>2007 2/16 (13%), 2010 4/24 (17%) failed visual inspection	Bate & Hess (2010)
Artemisinin monotherapies	Lagos, Nigeria	GQ, PQ			tab	r	2007 3/7 (43%), 2008 12/65 (18%), 2010 6/47 (13%) failed Minilab tests<br><br>2007 1/7 (14%), 2008 9/65 (14%), 2010 3/47 (6%) failed visual inspection	Bate & Hess (2010)
Artemether-lumefantrine	Burkina Faso	GQ			tab	C	0/4 failed Minilab assays	Tipke *et al.* (2008)
Artemether-lumefantrine	Cameroon	PQ	20/120	'Ajanta Pharma Ltd., India	tab	r	Tablets mottled, artemether 82%API. DHA impurity above limits	WHO (2011)
Artemether-lumefantrine	Ghana	PQ			tab	C	3/8 (38%) failed TLC and/or dissolution tests	Bate *et al.* (2008)
Artemether-lumefantrine	Ghana	F	'Coartem'		tab	S	Counterfeit packaging	Hope (2009), Kwei (2009), USP (2009b)

Artemether-lumefantrine	Ghana	PQ	20/120	'Jiangsu Yixing Forward Pharm Factory, China'	tab	r	Artemether 87.5% and lumefantrine 84.6 %API	WHO (2011)
Artemether-lumefantrine	Ghana	PQ	20/120	'Medreich PLC, India'	tab	r	Tablets mottled, lumefantrine 89.2% API	WHO (2011)
Artemether-lumefantrine	Kenya	PQ			tab	C	0/4 failed TLC and/or dissolution tests	Bate *et al.* (2008)
Artemether-lumefantrine	Kenya	PQ	20/120	'Novartis Pharmaceuticals Corporation, USA'	tab	r	High levels of artemether-related impurity	WHO (2011)
Artemether-lumefantrine	Madagascar	PQ	'Artefan'		tab	r	1/1 failed USP %API assay and/or dissolution testing	USP (2009a)
Artemether-lumefantrine	Nigeria	PQ			tab	C	1/7 (14%) failed TLC and/or dissolution tests	Bate *et al.* (2008)
Artemether-lumefantrine	Nigeria	F	'Lonart-DS'	'Bliss GVS Pharma Limited'	tab	S	60,000 tablets hidden in bangs, shoes and blankets, valued at N10 million, seized at Lagos International Airport. Detected with Raman spectroscope	Udoh (2010a)
Artemether-lumefantrine	Nigeria	F	'Lonart-DS 80/400'		tab	S	Imported from Hong Kong	Udoh (2010b)
Artemether-lumefantrine	Nigeria	F	'Persidon 20/120'		tab	S	Imported from Hong Kong	Udoh (2010b)
Artemether-lumefantrine	Nigeria	F	'Arthefantrin e'		tab	S	Imported from Hong Kong	Udoh (2010b)

Artemether-lumefantrine	Nigeria	PQ	20/120	'Ecomed Pharma Ltd., Nigeria'	tab	r	Tablets mottled, lumefantrine 85.9% API	WHO (2011)
Artemether-lumefantrine	Nigeria	PQ	20/120	'Jiangsu Yixing Forward Pharm Factory, China'	tab	r	Tablets with spots, artemether 89.7% API and lumefantrine 82.0 % API	WHO (2011)
Artemether-lumefantrine	Nigeria	PQ	20/120	'Jiangsu Yixing Forward Pharm Factory, China'	tab	r	Failed dissolution tests for lumefantrine	WHO (2011)
Artemether-lumefantrine	Nigeria	PQ	20/120	'Jiangsu Yixing Forward Pharm Factory, China'	tab	r	Artemether 85.5% API, lumefantrine 71.0% API. Failed dissolution tests for lumefantrine	WHO (2011)
Artemether-lumefantrine	Nigeria	PQ	20/120	'Jiangsu Yixing Forward Pharm Factory, China'	tab	r	Artemether 89.1% API, lumefantrine 87.3% API.	WHO (2011)
Artemether-lumefantrine	Nigeria	PQ	20/120	'Macleods pharmaceuticals Ltd.'	tab	r	Tablets mottled. No artemether detected, lumefantrine 92.2% API. Artemether-related impurities above limits	WHO (2011)

Artemether-lumefantrine	Nigeria	PQ	40/240	'May & Baker Nigeria PLC'	tab	r	Failed dissolution tests for lumefantrine	WHO (2011)
Artemether-lumefantrine	Nigeria	PQ	80/480	'Mekophar Chemical Pharmaceutical Joint Stock Company, Vietnam'	tab	r	Failed dissolution tests for lumefantrine	WHO (2011)
Artemether-lumefantrine	Nigeria	PQ	20/120	'Naxpar Lab Ltd, India'	tab	r	Failed uniformity of mass tests	WHO (2011)
Artemether-lumefantrine	Nigeria	PQ	20/120	'Naxpar Lab Ltd, India'	tab	r	Artemether 29.0% API, lumefantrine 81.5% API. Failed uniformity of mass and both artemether and lumefantrine dissolution tests	WHO (2011)
Artemether-lumefantrine	Rwanda	PQ			tab	C	0/3 failed TLC and/or dissolution tests	Bate *et al.* (2008)
Artemether-lumefantrine	Senegal	PQ	'Artefan'		tab	r	3/3 failed USP %API assay and/or dissolution testing	USP (2009a)
Artemether-lumefantrine	Tanzania	PQ			tab	C	0/1 failed TLC and/or dissolution tests	Bate *et al.* (2008)
Artemether-lumefantrine	Uganda	PQ			tab	C	2/9 (22%) failed TLC and/or dissolution tests	Bate *et al.* (2008)
Artemether-lumefantrine	Uganda	PQ	'Artefan'		tab	r	4/11 failed USP %API assay and/or dissolution testing	USP (2009a)

Artesunate + amodiaquine co-blistered	Cameroon	PQ	100/300	‘Adams Pharmaceuticals (ANHUI) Co Ltd, China’	tab	r	Artesunate 89.4% API	WHO (2011)
Artesunate + amodiaquine co-blistered	Cameroon	PQ	100/300	‘Adams Pharmaceuticals (ANHUI) Co Ltd, China’	tab	r	Artesunate related substances elevated	WHO (2011)
Artesunate + amodiaquine co-blistered	Cameroon	PQ	100/300	‘Adams Pharmaceuticals (ANHUI) Co Ltd, China’	tab	r	Artesunate 88.2% API. Artesunate related substances elevated	WHO (2011)
Artesunate + amodiaquine co-blistered	Cameroon	PQ	10/153.1	‘Plethico Pharmaceuticals Ltd, India’	tab	r	Amodiaquine 85.1% API	WHO (2011)
Artesunate + amodiaquine co-blistered	Ghana	PQ	100/300	‘Atlantic Pharmaceutical Ltd, Ghana’	tab	r	Artesunate 76.7% API. Artesunate related substances elevated. Amodiaquine uniformity of mass non-compliant	WHO (2011)
Artesunate + amodiaquine co-blistered	Ghana	PQ	50/153.1	‘Bliss GVS Pharma Ltd, India’	tab	r	Artesunate related substances elevated	WHO (2011)
Artesunate + amodiaquine co-blistered	Ghana	PQ	100/300	‘Danadams Pharmaceutical Industry Ltd, Ghana’	tab	r	Artesunate related substances elevated. Artesunate uniformity of mass non-compliant	WHO (2011)

Artesunate + amodiaquine co-blistered	Ghana	PQ	50/153.1	'Ipca Laboratories Ltd, India'	tab	r	Artesunate uniformity of mass non-compliant	WHO (2011)
Artesunate + amodiaquine co-blistered	Kenya	PQ	50/150	'Cosmos Ltd, Kenya'	tab	r	Artesunate related substances elevated	WHO (2011)
Artesunate + amodiaquine co-blistered	Madagascar	PQ	'Amosunate'		tab	r	2/2 failed USP %API assay and/or dissolution tests	USP (2009a)
Artesunate + amodiaquine co-blistered	Madagascar	PQ	'Falcimon'		tab	r	1/4 failed USP %API assay and/or dissolution tests	USP (2009a)
Artesunate + amodiaquine co-blistered	Nigeria	PQ	200/600	'Adams Pharmaceutical (ANHUI) Co Ltd, China'	tab	r	Artesunate 89.4% API. Artesunate related substances elevated. Artesunate dissolution non-compliant. Amodiaquine & artesunate uniformity of mass non-compliant	WHO (2011)
Artesunate + amodiaquine	Nigeria	PQ	50/150	'Adams Pharmaceutical (ANHUI) Co Ltd, China'	powder	r	Artesunate related substances elevated. Contaminant on TLC	WHO (2011)
Artesunate + amodiaquine co-blistered	Nigeria	PQ	100/300	'Baader Schulz Lab, India'	tab	r	Artesunate related substances elevated. Amodiaquine uniformity of mass non-compliant	WHO (2011)

Artesunate + amodiaquine co-blistered	Nigeria	PQ	50/153.1	'Baader Schulz Lab, India'	tab	r	Amodiaquine 115% API. Artesunate related substances elevated. Artesunate uniformity of mass non-compliant. Contaminant on TLC	WHO (2011)
Artesunate + amodiaquine co-blistered	Nigeria	PQ	50/150	'Madras Pharmaceuticals, India'	tab	r	Artesunate related substances elevated. Contaminant on TLC	WHO (2011)
Artesunate + amodiaquine	Nigeria	PQ	50/150	'Madras Pharmaceuticals, India'	tab	r	Artesunate related substances elevated. Contaminant on TLC	WHO (2011)
Artesunate + amodiaquine co-blistered	Nigeria	PQ	50/153.1	'Saga Laboratories Ltd, India'	tab	r	Artesunate failed disintegration and dissolution tests. Artesunate related substances elevated.	WHO (2011)
Artesunate + amodiaquine co-blistered	Nigeria	PQ	50/153.1	'Saga Laboratories Ltd, India'	tab	r	Artesunate failed dissolution tests. Artesunate related substances elevated.	WHO (2011)
Artesunate + amodiaquine co-blistered	Nigeria	PQ	50/200	'Swiss Pharma Nigeria Ltd, Nigeria'	tab	r	Artesunate related substances elevated. Contaminant on TLC	WHO (2011)
Artesunate + amodiaquine co-blistered	Senegal	PQ	'Camoquin'		tab	r	2/2 failed USP %API assay and/or dissolution tests	USP (2009a)
Artesunate + amodiaquine co-blistered	Senegal	PQ	'Falcimon'		tab	r	4/15 (27%) failed USP %API assay and/or dissolution tests	USP (2009a)

Artesunate + amodiaquine co-blistered	Senegal	PQ	'Larimal'		tab	r	1/1 failed USP %API assay and/or dissolution tests	USP (2009a)
Artesunate + amodiaquine co-blistered	Uganda	PQ	'Duact'		tab	r	2/2 failed USP %API assay and/or dissolution tests	USP (2009a)
Artesunate + amodiaquine co-blistered	Uganda	PQ	'Larimal'		tab	r	5/5 failed USP %API assay and/or dissolution tests	USP (2009a)
Artesunate + amodiaquine coformulated	Cameroon	PQ	50/200	'Kamala Overseas, India'	tab	r	Artesunate 82.5% API. Artesunate failed dissolution tests	WHO (2011)
Artesunate + amodiaquine coformulated	Cameroon	PQ	50/200	'Kamala Overseas, India'	tab	r	Artesunate 73.1% API. Artesunate failed dissolution tests	WHO (2011)
Artesunate + amodiaquine coformulated	Cameroon	PQ	50/200	'Kamala Overseas, India'	tab	r	Artesunate 72.4% API. Contaminant on TLC	WHO (2011)
Artesunate + amodiaquine co-blistered	Madagascar	PQ	'Coasucam'		tab	r	1/11 (9%) failed USP %API assay and/or dissolution tests	USP (2009a)
Artesunate + amodiaquine coformulated	Nigeria	PQ	100/306.2	'Emzor Pharma Ind. Ltd, Nigeria'	caplet	r	Artesunate 87.2% API. Contaminant on TLC	WHO (2011)
Artesunate + amodiaquine coformulated	Nigeria	PQ	100/306.2	'Emzor Pharma Ind. Ltd, Nigeria'	caplet	r	Artesunate failed dissolution tests. Contaminant on TLC	WHO (2011)
Artesunate + amodiaquine coformulated	Nigeria	PQ	100/306.2	'Emzor Pharma Ind. Ltd, Nigeria'	caplet	r	Artesunate 82.5% API. Contaminant on TLC	WHO (2011)
Artesunate + amodiaquine coformulated	Nigeria	PQ	50/150	'Emzor Pharma Ind. Ltd, Nigeria'	caplet	r	Artesunate 87.8% API. Contaminant on TLC	WHO (2011)

Artesunate + amodiaquine coformulated	Nigeria	PQ	200/600	'Mercury Laboratories Ltd, India'	tab	r	Artesunate failed dissolution tests	WHO (2011)
Artesunate + amodiaquine coformulated	Nigeria	PQ	50/150	'Rajat Pharmachem Ltd, India'	powder	r	Amodiaquine 76.4% API. Contaminant on TLC	WHO (2011)
Artesunate + Sulphadoxine-pyrimethamine co-blistered	Senegal	PQ	'Co-arinate'		tab	r	1/3 (33%) failed USP %API assay and/or dissolution tests	USP (2009a)
Artesunate + mefloquine co-blistered	Senegal	PQ	'Artequin'		tab	r	3/3 failed USP %API assay and/or dissolution tests	USP (2009a)
Dihydroartemisinin-piperaquine	Kenya	F	'Duo-Cotecxin'	'Beijing Holley Cotec Pharmaceuticals Company Ltd.'	tab	W	Counterfeit	Kenya Pharmacy Board (2007), Anon (2007)
ACTs	Accra, Ghana	GQ, PQ			tab	r	2007 3/8 (38%), 2010 1/10 (10%) failed Minilab tests<br>2007 1/8 (13%), 2010 0/10 (0%) failed visual inspection	Bate & Hess (2010)
ACTs	Lagos, Nigeria	GQ, PQ			tab	r	2007 1/7 (14%), 2008 1/5 (20%), 1/17 (6%) failed Minilab tests<br><br>2007 1/7 (14%), 2008 0/5 (0%), 2010 0/17 (0%) failed visual inspection	Bate & Hess (2010)

References

Abdi YA, Rimoy G, Ericsson O, Alm C, Massele AY: **Quality of chloroquine preparations marketed in Dar es Salaam, Tanzania**. *Lancet* 1995, **346**: 1161.

Alfadl AA, Abdoon S, Elamin M, Elnabi NG: **Quality of Antimalarial Drugs in Sudan: Results of Post-Marketing Surveillance**. *Sudanese Journal of Public Health* 2006, **1**: 108-11. Available: http://indexmedicus.afro.who.int/iah/fulltext/quality-antimalarial.pdf. Accessed 15th April 2011.

Aina BA, Tayo F, Taylor O: **Quality of chloroquine dosage forms in Lagos State General Hospitals.** *Nigeria. J. Pharm. Pharmacol,* 2007: Suppl 1. A44.

Amin AA, Snow RW, Kokwaro GO: **The quality of sulphadoxine-pyrimethamine and amodiaquine products in the Kenyan retail sector.** *J. Clin. Pharm. Ther.* 2005, **30**: 559-65.

Amin AA, Kokwaro GO: **Antimalarial drug quality in Africa.** *J. Clin. Pharm. Ther.* 2007, **32**: 429-440.

Anon: **Fake quinine tablets alert in Rwanda**. Business Action to Stop Counterfeiting and Piracy, 1st April 2001. Available at: http://88.208.193.7/bascap/article.php?articleid=344. Accessed 12 October 2011.

Anon: **GlaxoSmithKline case study** (7th April 2003). Judicial Protection of IPR in China, 2003a. Available: http://www.chinaiprlaw.com/english/news/news14.htm. Accessed 12 March 2011.

Anon: **Counterfeiting abroad and home: British Pharmaceutical Conference.** *Pharm. J* 2003b, **271**: 453–4.

Anon: **Belgian customs seize record haul of fake pills, from India.** 2 October 2008. Available at http://www.protectmymeds.com/site/news_room/belgian_customs_seize_record_haul_of_fake_pills_from_india. Accessed 12 October 2011.

Anon: **Malaria drugs recalled in Kenya.** *BBC News*, 2007. Available: http://news.bbc.co.uk/1/hi/world/africa/6951586.stm. Accessed 12 March 2011.

Antony PGR, Temu-Justin M: **The quality of drugs manufactured in Tanzania.** *East Cent. Afr. J. Pharm. Sci.* 1999, **2**: 45-49.

Arrow KJ: **Fake malaria drugs in Tanzania.** *Marketletter*, 20 February 2001.

Atemnkeng MA, De Cock K, Plaizier-Vercammen J: **Quality control of active ingredients in artemisinin derivative antimalarials within Kenya and DR Congo**. *Trop. Med. Int. Hlth.* 2007, 12: 68–74.

Barbereau S: **Guinea: faux medicaments.** *E-med* Jan 11, 2006. Available http://www.essentialdrugs.org/emed/archive/200601/msg00038.php. Accessed 12 October 2011.

Basco LK, Ringwald P, Manene AB, Chandenier J: **False chloroquine resistance in Africa**. *Lancet* 1997, **350**: 224.

Basco LK: **Molecular epidemiology of malaria in Cameroon. XIX. Quality of antimalarial drugs used for self-medication.** *Am. J. Trop. Med. Hyg.* 2004, **70**: 245–50.

Bate R, Coticelli P, Tren R, Attaran A. **Antimalarial drug quality in the most severely malarious parts of Africa - a six country study.** *PLoS One* 2008, **3**: e2132.

Bate R, Hess K: **Anti-malarial drug quality in Lagos and Accra - a comparison of various quality assessments.** *Malar. J.* 2010, **9:** 157

Bogere H, Nafula J: **Fake quinine on market.** *Daily Monitor,* 10th May 2007.

Cockburn R, Newton PN, Agyarko EK, Akunyili D, White NJ . **The global threat of counterfeit drugs: why industry and governments must communicate the dangers.** *PLoS Medicine* 2005, **2**: e100.

Deisingh, AK: **Pharmaceutical Counterfeiting.** *Analyst* 2005, **130**: 271–279.

Economist Intelligence Unit: **World Pharma: A Potential Massacre.** 14th December 2009. Available: http://viewswire.eiu.com/index.asp?layout=ib3Article&article_id=1685058953&pubtypeid=1152462500&country_id=1510000351&IBNL=true&rf=0. Accessed Jan 21 2010.

Edike T: **NAFDAC raids fake drug label printing house.** *Vanguard (Lagos).* 12 December 2003.

Erhun WO, Babalola OO, Erhun MO: **Drug regulation and control in Nigeria: The challenge of counterfeit drugs.** *J. Health Pop. Develop. Countries* 2001, **4**: 23–34.

Esimone CO, Okoye FB, Onah BU, Nworu CS, Omeje EO: **In vitro bioequivalence study of nine brands of artesunate tablets marketed in Nigeria**. *J. Vector Borne Dis*. 2008a, **45**: 60-5.

Esimone CO, Omeje EO, Okoye FB, Obonga WO, Onah BU: **Evidence for the spectroscopic determination of artesunate in dosage form.** *J. Vector Borne Dis.* 2008b, **45**: 281-6.

Gaudiano MC, Antoniella E, Bertocchi P, Valvo L: **Development and validation of a reversed-phase LC method for analysing potentially counterfeit antimalarial medicines.** *J. Pharm. Biomed. Anal.* 2006, **42**: 132-5.

Gaudiano MC, Di Maggio A, Cocchieri E, Antoniella E, Bertocchi P, Alimonti S, Valvo L.: **Medicines informal market in Congo, Burundi and Angola: counterfeit and sub-standard antimalarials.** *Malar. J.* 2007, **6**: 22.

Ghana News Agency: **Pharmacia withdraws metakelfin in white sachets. Available at:**

17 Feb 2002. http://www.modernghana.com/news/20469/1/pharmacia-withdraws-metakelfin-in-white-sachets.html. Accessed 12 October 2011.

Ghana News Agency: **Ghana blames Chinese companies for dumping fake anti-malaria drugs.** *Africa Review* 23 June 2011. Available at: http://www.africareview.com/News/Ghana+blames+Chinese+companies+for+fake+malaria+drugs/-/979180/1187694/-/pasi7j/-/login. Accessed 12 October 2011.

Ghana News Agency: **FDB recalls Counterfeit and Substandard Anti-Malarial Medicines.** Ghana News Agency 8th November 2010. Available at http://www.ghanaweb.com/GhanaHomePage/health/artikel.php?ID=197020#. Accessed 12 October 2011.

Hebron Y, Tettey JNA, Pournamdari M, Watson DG: **The chemical and pharmaceutical equivalence of sulphadoxine/pyrimethamine tablets sold on the Tanzanian market.** *J. Clin. Pharm. Ther.* 2005, **30**: 575-581.

Hope KE: **Beware of fake Co-Artem malaria tabs on the market.** *The Ghanaian Times* July 10th 2009.

Idowu OA, Alapara SB, Lasisi AA: **Assessment of quality of chloroquine tablets sold by drug vendors in Abeokuta, Nigeria.** *Tanz. Health Res. Bull.* 2006, **8**: 45–46.

Ioset JR, Kaur H: **Simple field assays to check quality of current artemisinin-based antimalarial combination formulations**. *PLoS One* 2009, **4**: e7270.

Jähnke RWO: **Counterfeit medicines and the GPHF-Minilab for rapid drug quality verification.** *Pharm. Ind.* 2004, **66**: 1187–1193.

Jande MB, Ngassapa O, Kibwage IO: **Quality of Sulfadoxine/Pyrimethamine tablets marketed in Dar es Salaam, Tanzania.** *East Cent. Afr. J. Pharm. Sci.* 2000, **3**: 20-24.

Kaur H, Goodman C, Thompson E, Thompson KA, Masanja MI, Kachur SP, Abdulla S.: **A Nationwide Survey of the Quality of Antimalarials in Retail Outlets in Tanzania.** *PLoS One* 2008, **3**: 10.

Kayumba PC, Risha PG, Shewiyo D, Msami A, Masuki G, Ameye D, Vergote G, Ntawukuliryayo JD, Remon JP, Vervaet C: **The quality of essential antimicrobial and antimalarial drugs marketed in Rwanda and Tanzania: influence of tropical storage conditions on in vitro dissolution.** *J. Clin. Pharm. Ther*. 2004, 29: 331-8.

Kibwage IO, Thuranira JK, Gathu L, Githiga LM, Nguyo JM, Ngugi JK, King'ondu O: **Drug quality control work in the drug analysis and research unit: observation during 1991-1995.** *East Cent. Afr. J. Pharm. Sci.* 1999, **2**: 32-36.

Kibwage IO, Ngugi JK: **Sulphadoxine/pyrimethamine tablet products on the Kenyan market: quality concerns.** *East Cent. Afr. J. Pharm. Sci.* 2000, **3**: 14–9.

Koch E, Simmm M, Wech M: **La mafia des faux medicaments.** *Courrier Int.* 1994, **204**: 36.

Kron MA: **Substandard primaquine phosphate for US Peace Corps personnel.** *Lancet* 1996, **348**: 1453–4.

Kwei R: **Counterfeit drugs threaten to roll back the gains**. *Eyes on Malaria (AMREN Magazine)* 2009. Available: http://www.eyesonmalaria.org/fourthedition27.html. Accessed 12 March 2011.

Legris, C : **La détection des médicaments contrefaits par investigation de leur authenticité. Étude pilote sur le marché pharmaceutique illicite de Côte d'Ivoire.** Thèse pour le diplôme d'état de docteur en pharmacie soutenus à la Faculté de Pharmacie Nancy I ; 2005. Available at: http://www.remed.org/html/theses.html. Accessed 12 October 2011.

Mahmoud BM, Ali HM, Homeida MMA, Bennett JL: **Bioequivalence of five chloroquine brands marketed in Sudan**. *Int. Pharm. J.* 1994, **8**: 164–167.

Minzi OM, Moshi MJ, Hipolite D, Massele AY, Tomson G, Ericsson O, Gustafsson LL: **Evaluation of the quality of amodiaquine and sulphadoxine/pyrimethamine tablets sold by private wholesale pharmacies in Dar Es Salaam Tanzania.** *J. Clin. Pharm. Ther.* 2003, **28**: 117-22.

Minzi OM, Massele A, Justin-Temu M, Ericsson O, Gustafsson LL. **Existence of antimalarial formulations with low bioavailability in Tanzania.** *Trop. Doct.* 2006, **36:** 93-7.

Mugabe D: **Fake drugs hit Ugandan market.** *The New Vision* 7th September 2009.

Murphy SA, Mberu E: **A study of the comparative bioavailability of pyrimethamine-sulfadoxine obtained from two oral preparations.** *East Afr. Med. J.* 1994, **71**: 328-9.

Ndomondo-Sigonda M: **Counteracting counterfeiting: strategies for improving the integrity of the medicinal marketplace in Tanzania.** 2005. Available at http://www.msh.org/seam/reports/fip-cairo_2005/57_counteracting.pdf. Accessed 12 October 2011.

Newton PN, Green MD, Fernández FM, Day NJP, White NJ: **Counterfeit anti-infective medicines**. *Lancet Infect. Dis.* 2006a, **6**: 602-613.

Newton PN, McGready R, Fernández F, Green MD, Sunjio M, Bruneton C, Phanouvong S, Millet P, Whitty CJ, Talisuna AO, Proux S, Christophel EM, Malenga G, Singhasivanon P, Bojang K, Kaur H, Palmer K, Day NPJ, Greenwood BM, Nosten F, White NJ: **Manslaughter by fake artesunate in Asia –will Africa be next ?** *PLoS Medicine* 2006b, **3**: e197.

Nsimba SE, Aden-Abdi Y, Rimoy G, Massele AY, Alm C, Ericsson O, Gustafsson LL:, **Comparative in vitro and in vivo study of a sugar-coated chloroquine preparation marketed in Tanzania versus an ordinary brand.** *J. Clin. Pharm. Ther.* 2001, **26**: 43-8.

Odeniyi MA, Adegoke OA, Adereti RB, Odeku OA, Itiola OA: **Comparative analysis of eight brands of sulfadoxine-pyrimethamine tablets.** *Trop. J. Pharm. Res.* 2003, **2:** 161-167.

Odunfa OO, Adegoke OA, Onaga IC: **Pharmaceutical equivalence of some commercial samples of artesunate and amodiaquine tablets sold in Southwestern Nigeria.** *Trop. J. Pharm. Res.* 2009, **8**: 491-499.

Ofori-Kwakye K, Y Asantewaa, O Gaye: **Quality of artesunate tablets sold in pharmacies in Kumasi, Ghana.** *Trop. J. Pharm. Res.* 2008, **7**: 1179-1184.

Ogwal-Okeng JW, Okello DO, Odyek O: **Quality of oral and parenteral chloroquine in Kampala.** *East Afr. Med. J.* 1998, **75**: 692-4.

Ogwal-Okeng JW, Owino E, Obua C: **Chloroquine in the Ugandan market fails quality test: a pharmacovigilance study.** *Afr. Health Sci.* 2003, **3**: 2–6.

Onwujekwe O, Kaur H, Dike N, Shu E, Uzochukwu B, Hanson K, Okoye V, Okonkwo P: **Quality of anti-malarial drugs provided by public and private healthcare providers in south-east Nigeria.** *Malar. J.* 2009, **8**: 22.

Oyeniran M: **NAFDAC seizes N3M malaria drugs**. *Nigeria Tribune* 28 Sept 2006. Available: http://www.tribune.com.ng/28092006/news/news13.html. Accessed 3 Nov

2006.

Oyeniran M: **NAFDAC seizes N10M malaria drugs.** *Nigeria Tribune* 4 April 2007.

Ogundipe S: **Nigeria: Nafdac Seizes N32 Million Fake Anti-Malarial Drugs** 2 June 2009. Available at http://allafrica.com/stories/200906020015.html. Accessed 12 October 2011.

Reidenberg MM, BA Conner: **Counterfeit and substandard drugs.** *Clin. Pharmacol. Ther.* 2001, **69**: 189-193.

Rimoy GH, Moshi MJ, Massele AY: **Comparative bioavailability of oral sugar-coated and plain formulation of chloroquine phosphate marketed in Tanzania.** *Trop. Doct.* 2002, **32**: 15-7.

Risha PG, Shewiyo D, Msami A, Masuki G, Vergote G, Remon JP: **In vitro evaluation of the quality of essential drugs on the Tanzanian market.** *Trop. Med. Int. Hlth.* 2002, **7**: 701-7.

Risha PG, Msuya Z, Clark M, Johnson K, Ndomondo-Sigonda M, Layoff T: **The use of Minilabs to improve the testing capacity of regulatory authorities in resource limited settings: Tanzanian experience.** *Health Policy* 2008, **87**: 217-22.

Sesay MM: **Fake drugs – a new threat of health care delivery.** *Africa Health* June/July 1988, 13–15.

Shakoor O, Taylor RB, Behrens RH: **Assessment of the incidence of substandard drugs in developing countries.** *Trop. Med. Int. Hlth.* 1997, **2**: 839-845.

Shekighenda L: **TFDA halts sale, use of metakelfin.** 1st April 2009. *IPPmedia*. Available at: http://www.ippmedia.com/ . Accessed 12 October 2011.

Smine A., Diouf K, Blum NL: **USPDQI Antimalarial Drug Quality in Senegal 2002**. Submitted to the U.S. Agency for International Development by the United States Pharmacopeia Drug Quality and Information Program. Rockville, MD: United States Pharmacopeia. Available at: http://pdf.usaid.gov/pdf_docs/PNACW987.pdf. Accessed 12 October 2011.

Sowunmi A, Salako LA, Ogunbona FA: **Bioavailability of sulphate and dihydrochloride salts of quinine.** *Afr. J. Med. Sci.* 1994, **23**: 275-8.

Taylor RB, Shakoor O, Behrens RH, Everard M, Low AS, Wangboonskul J, Reid RG, Kolawole JA: **Pharmacopoeial quality of drugs supplied by Nigerian pharmacies.** *Lancet* 2001, **357**: 1933–1936.

ten Ham M: **Counterfeit drugs: implications for health.** *Adverse Drug React. Toxicol. Rev.* 1992, **11**: 59-65.

Thoithi GN, Abuga KO, Nguyo JM, Mukindia G, Kingondu O, Ngugi JK, Kibwage IO: **Drug quality control in Kenya: observation in drug analysis and research unit during the period 1996-2000.** *East Cent. Afr. J. Pharm. Sci.* 2002, **5**: 28-32.

Tipke M, Diallo S, Coulibaly B, Störzinger D, Hoppe-Tichy T, Sie A, Müller O: **Substandard anti-malarial drugs in Burkina Faso**. *Malar. J.* 2008, **7:** 95.

The Pharmacy and Poisons Board, Republic of Kenya Ministry of Health. 2007. **Public Alert**. Available: http://www.pharmacyboardkenya.org. Accessed 12 March 2011.

Udoh F: **NAFDAC impounds N10 Million Fake Malaria Drugs.** *AllAfrica.com.* 21 Jan 2010a.

Udoh F: **Nigeria: NAFDAC impounds consignments of fake drugs valued at N500 Million.** *AllAfrica.com*. 10 June 2010b.

United States Pharmacopeia: **Survey of the quality of selected antimalarial medicines circulating in Madagascar, Senegal, and Uganda**. United States Pharmacopeial Convention, 2009a. Available: http://www.usaid.gov/our_work/global_health/hs/publications/qamsa_report_1109.pdf.

Accessed 12 March 2011.

United States Pharmacopeia: **Counterfeit antimalarial drug discovered in Ghana with aid of USP Drug Quality and Information Program.** Press release, USP, 2009b. Available at www.uspdqi.org. Accessed 12 March 2011.

Wolff JC, Thomson LA, Eckers C: **Identification of the 'wrong' active pharmaceutical ingredient in a counterfeit Halfan drug product using accurate mass electrospray ionisation mass spectrometry, accurate mass tandem mass spectrometry and liquid chromatography/mass spectrometry**. *Rapid Comm. Mass. Spect.* 2003, **17**: 215–221.

World Health Organization: **La qualité des médicaments sur le marché pharmaceutique africain - Étude analytique dans trois pays: Cameroun, Madagascar, Tchad.** Série de recherche, No. 18. WHO/DAP/95.3. 1995. Available at: http://apps.who.int/medicinedocs/collect/medicinedocs/pdf/s2212f/s2212f.pdf. Accessed 12 October 2011.

World Health Organization: **The quality of antimalarials – A study in selected African countries.** WHO/EDM/PAR/2003.4. Geneva. 2003. Available at: http://www.who.int/medicinedocs/pdf/s4901e/s4901e.pdf. Accessed 12 October 2011.

World Health Organization: **Survey of the quality of selected antimalarial medicines circulating in six countries of sub-Saharan Africa**. World Health Organization.

Geneva, 2011. WHO/EMP/QSM/2011.1. Available: http://www.who.int/medicines/publications/WHO_QAMSA_report.pdf. Accessed 12 March 2011.
