# Supplementary material for: Poor quality vital anti-malarials in Africa - an urgent neglected public health priority
Source: Malar J. 2011 Dec 13;10:352. doi: 10.1186/1475-2875-10-352 (PMC3262771; doi:10.1186/1475-2875-10-352)
Supplement: Additional file 2 — Summary of packaging, chemical and botanical analysis of anti-malarial samples. Results for tablets with AI chemical content < 90% or > 110% relative to the stated dose in red. [file 1475-2875-10-352-S2.PDF]

Additional File 2. Summary of packaging, chemical and botanical analysis of antimalarial samples. Results for tablets with API chemical content <90% or > 110% relative to the stated dose in red

**Sample Code**<br>**Sample location**<br>**Formulation**<br>**Classification**	**Packaging**	**Batch No**<br>**Mfg Date**<br>**Exp Date**<br>**{Putative Types}**	**Mass spectrometry**	**HPLC-UV mg/tablet**	**XRD**	**Botany**
**A. Counterfeit artesunate collected in Cameroon. All stated to contain 50mg artesunate/tablet**						
Cameroon S5/07<br>12x50mg white tablets /blister with packet and leaflet. Collected July 2007<br><br>Counterfeit	'Artesunat' stated to be manufactured by 'Mekophar Chemical Pharmaceutical Joint-Stock Company'<br>'Mekophar' hologram on packet<br>NAFDAC 04-3397<br>Reg. N^o/N^o VNA-2892-99<br>Bar code 8934574010511<br>'Marketed by: Neros Pharmaceuticals Ltd., Lagos Nigeria'	0170805<br>08/2005<br>08/2008	chloroquine$^{\alpha, \beta}$, sucrose$^{\alpha}$	56.0, 58.0 mg/tablet chloroquine	starch (C), organic compounds (C)	Fungal spores, plant cells, pollen grains of *Typha angustifolia*, charcoal, black chips
Cameroon S5 1/08<br>12x50mg white tablets /blister with packet and leaflet. Collected January 2008<br><br>Counterfeit	'Artesunat' stated to be manufactured by 'Mekophar Chemical Pharmaceutical Joint-Stock Company'<br>'Mekophar' hologram on packet<br>NAFDAC REG N^o : 04-3397<br>Reg. N^o/N^o VNA-2892-99<br>Bar code 8934574010511	06027FX<br>07/2006<br>07/2009	chloroquine$^{\alpha, \beta}$<br>no sucrose	--------^{IN}	starch (M), dextrose (A), talc (T)	Fungal spores, fern spores, *Pinus* pollen grain, traces of charcoal, black chips, cellular and amorphous organic debris

	Marketed by: Neros Pharmaceuticals Ltd., Lagos Nigeria					
Cameroon S5 2/08 12x50mg white tablets /blister with packet and leaflet. Collected January 2008<br><br>Counterfeit	'Artesunat' stated to be manufactured by 'Mekophar Chemical Pharmaceutical Joint-Stock Company' and 'Marketed by: Neros Pharmaceuticals Ltd., Lagos Nigeria'<br>'Mekophar' hologram on packet<br>NAFDAC REG N^o : 04-3397<br>Reg. N^o/N^o VNA-2892-99<br>Bar code 8934574010511	06027FX<br>07/2006<br>07/2009	chloroquine$^{\alpha, \beta}$<br>no sucrose	--------^{IN}	starch (M), dextrose (A), talc (T)	Fungal spores, fern spores, *Ulmus* pollen grain, cellular and amorphous debris
Cameroon S5 3/08 12x50mg white tablets /blister with packet and leaflet. Collected January 2008<br><br>Counterfeit	'Artesunat' stated to be manufactured by 'Mekophar Chemical Pharmaceutical Joint-Stock Company' and 'Marketed by: Neros Pharmaceuticals Ltd., Lagos Nigeria'<br>'Mekophar' hologram on packet<br>NAFDAC REG N^o : 04-3397<br>Reg. N^o/N^o VNA-2892-99<br>Bar code 8934574010511	06027FX<br>07/2006<br>07/2009	chloroquine$^{\alpha, \beta}$<br>no sucrose	--------^{IN}	starch (M), dextrose (A), talc (T)	Fungal spores (~20 µm), rare Asteraceae (daisies), Chenopodiaceae and a folded tricolporate pollen grain, fungal hyphae, cellular and amorphous debris
Cameroon S5 4/08 12x50mg white tablets/blister with packet and leaflet.	'Artesunat' stated to be manufactured by 'Mekophar Chemical Pharmaceutical Joint-	06027FX<br>07/2006<br>07/2009	chloroquine$^{\alpha, \beta}$<br>no sucrose	--------^{IN}	starch (M), dextrose (A), talc (T)	Fungal spores, pollen grain?, charcoal, black chips, cellular and amorphous organic debris, plant cellular material

Collected January 2008<br><br>Counterfeit	Stock Company' and 'Marketed by: Neros Pharmaceuticals Ltd., Lagos Nigeria'<br>'Mekophar' hologram on packet<br>NAFDAC REG N^{o} : 04-3397<br>Reg. N^{o}/N^{o} VNA-2892-99<br>Bar code 8934574010511					(up to ~160 µm)
Cameroon S5 5/08 12x50mg white tablets /blister with packet and leaflet. Collected January 2008<br><br>Counterfeit	'Artesunat' stated to be manufactured by 'Mekophar Chemical Pharmaceutical Joint-Stock Company' and 'Marketed by: Neros Pharmaceuticals Ltd., Lagos Nigeria'<br>'Mekophar' hologram on packet<br>NAFDAC REG N^{o} : 04-3397<br>Reg. N^{o}/N^{o} VNA-2892-99<br>Bar code 8934574010511	06027FX<br>07/2006<br>07/2009	chloroquine$^{\alpha, \beta}$<br>no sucrose	--------[IN]	starch (M), dextrose (A), talc (T)	Fungal spores, charcoal, black chips, Myrtaceae and Poaceae (grass) pollen, cellular and amorphous organic debris, plant cellular material
Lao 07/24-1, 07/24-2, 07/24-3, 07/24-4 12x50mg white tablets /blister with packet and leaflet<br><br>Genuine	'Artesunat' stated to be manufactured by 'Mekophar Chemical Pharmaceutical Joint-Stock Company'<br>No hologram<br>SDK/Reg.No: VNB-2339-04<br>Bar code 8934574010245	06004FN<br>07/08/06<br>08/2009	artesunate	52.0, 51.0, 52.0, 52.0, 52.0 mg/tablet	organic compounds (A), starch (M), talc (T)	Very rare charcoal, ?*Ulmus*, Cyatheaceae fern spores, Poaceae, Brassicaceae, Myrtaceae, Chenopodiaceae,

Lao 07/25-1, 07/25-2, 07/25-3, 07/25-4<br><br>12x50mg white tablets /blister with packet and leaflet<br>Genuine	'Artesunate' stated to be manufactured by 'Mediplantex 358 Giai Phong Road – Hanoi- Viet Nam'<br>No hologram<br>Reg.No.<br>VNA – 2820-05<br>Myanmar Reg. No: 0907A7304<br>Bar code 893504100035<br>Packet circular seal	061106<br>31/11/06<br>11 2009	artesunate	50.5, 52.5, 48.0 mg/tablet	starch (A), talc (M)	Charcoal (some wood derived), Poaceae, *Pinus, Betula,* Palmae, *Trema, Alnus, Fraxinus,* Cyperaceae (sedges), Chenopodiaceae pollen, Cyatheaceae and Hymenophyllaceae fern spores
Thai/Burma border<br>S 66<br>12x50mg white tablets /blister with packet and leaflet<br><br>Genuine	'Artesunat' stated to be manufactured for 'AA Medical Products Ltd., by Mekophar, Vietnam'<br>MYN Reg.No. : R0906A3639	07038FX<br>09/2007<br>09/2010	artesunate	52.5 mg/tablet	organic compounds (A), starch (M), talc (T)	Poaceae with fungal spores inside, *Pinus, Typha*, Apiaceae (carrot family), Asteraceae, Chenopodiaceae pollen
Thai/Burma border<br>S 67<br>12x50mg white tablets /blister with packet and leaflet<br><br>Genuine	'Artesunate' stated to be manufactured for 'AA Medical Products Ltd., by Mekophar, Vietnam'<br>MYN Reg.No. : 0406A3639	06038FX<br>09/2006<br>09/2009	artesunate	51.5 mg/tablet	organic compounds (A), starch (M), talc (T)	Black chips, hyaline "cysts", Poaceae, ?Sapotaceae pollen
Mek 10/03, 10/05<br>Genuine, direct from manufacturer<br>12x50mg white tablets /blister with packet and leaflet<br><br>Genuine	'Artesunat' stated to be manufactured by 'Mekophar Chemical Pharmaceutical Joint-Stock Co.' and marketed by : 'NEROS PHARMACUETICALS LTD., LAGOS, NIGERIA.'<br>'Neros MKP' hologram	10005FX<br>25/03/10<br>25/03/13	artesunate	artesunate 51.0, 50.0, 49.0 and 51.0, 51.0, 52.0 mg/tablet	-------	---------------

**B. Counterfeit dihydroartemisinin (DHA) collected in Kenya and Nigeria.** All samples stated to contain 60mg DHA. All stated to be manufactured by 'Jiaxing Nanhu Pharmaceutical Co. Ltd. Jiaxing City, under license of Beijing Holley-Cotec Pharmaceuticals (PR China)' with stated trade name 'Cotecxin'						
Kenya 07/01, Genuine<br>8x60mg white tablets/blister with packet and leaflet<br><br>Genuine	Tablet diameter 9.22 mm<br>& thickness=3.98 mm	MFD 10112006; EXP 11/2009; LOT 031106	dihydroartemisinin$^{\alpha, \beta}$	60.6 mg/tablet dihydroartemisinin	organic compounds (C), lactose (M), glucose (M), starch (M)	Charcoal fragments, isolated plant cells, very rare fungal spores and hyphae, *Picea*, Malvaceae (probably *Hibiscus* spp.), Moraceae (cannabis family)
Kenya 07/02<br>8x60mg white tablets/blister with packet and leaflet<br><br>Counterfeit	Tablet diameter= 9.14 mm & thickness=5.27 mm. Differences in colour of packaging	MFD 10042006; EXP 04/2009; LOT 030406	No API detected	No dihydroartemisinin detected	starch (A), dolomite (M), calcite (M), quartz (M)	Abundant fine charcoal, rare fungal hyphae, occasional fungal cellular clusters and at least 4 different types of abundant, relatively large (up to 37 µm), mainly thick walled, brown fungal spores, totaling over 3,300 on the 22 slides (i.e. ~ 1000 grains per tablet). *Pinus*, Asteraceae, Chenopodiaceae, Fagaceae, *Quercus*?, Rubiaceae
Private hospital, Nigeria [1]<br>8x60mg white tablets/blister<br><br>Counterfeit	-------	MFD 2003;<br>EXP November 2005<br>Lot Number 02110	acetaminophen $^{\beta}$	No dihydroartemisinin detected (LSHTM)	---------	---------
**C. Counterfeit halofantrine collected in West Africa and China. Tablets stated to contain 250mg halofantrine**						
BN 694 Genuine<br>6 tablets/blister<br>Direct from manufacturer<br><br>Genuine	'Halfan' stated to be manufactured by 'SmithKline Beecham Laboratoires Pharmaceutiques'	694<br>Mfg Apr02<br>Exp Apr05<br>{Genuine}	halofantrine$^{\alpha, \beta}$	250 mg/tablet halofantrine$^{\gamma}$	---------	-----------------------------------

2007 Sierra Leone<br>6 tablets/blister<br><br>Counterfeit	‘Halfan’ stated to be manufactured by ‘SmithKline Beecham Laboratoires Pharmaceutiques’	550<br>Mfg Jun99<br>Exp Jun02<br>{A}	artemisinin$^{\alpha, \beta}$	25.0 mg/tablet artemisinin	---------	-----------------------------------
2008 Sierra Leone<br>6 tablets/blister<br><br>Counterfeit	‘Halfan’ stated to be manufactured by ‘SmithKline Beecham Laboratoires Pharmaceutiques’	605<br>Mfg May00<br>Exp May03<br>{A}	artemisinin$^{\alpha, \beta}$	21.0 mg/tablet artemisinin	---------	-----------------------------------
2015 Nigeria<br>6 tablets/blister<br><br>Counterfeit	‘Halfan’ stated to be manufactured by ‘SmithKline Beecham Laboratoires Pharmaceutiques’	613<br>Mfg Jan00<br>Exp Jan03<br>{A}	artemisinin$^{\alpha, \beta}$	35.0 mg/tablet artemisinin	---------	-----------------------------------
4020 Nigeria<br>6 tablets/blister<br><br>Counterfeit	‘Halfan’ stated to be manufactured by ‘SmithKline Beecham Laboratoires Pharmaceutiques’ Same blister design (apart from use of different batch data), same rudimentary ‘GSK’ holographic label and same leaflet as 4023. However, blister strip is not from the same tooling as 4023.	709<br>Mfg Jun02<br>Exp Jun05<br>{D}	acetaminophen$^{\alpha, \beta}$	317 mg/tablet acetaminophen	---------	-----------------------------------

4021 Nigeria<br>6 tablets/blister<br><br>Counterfeit	'Halfan' stated to be manufactured by 'SmithKline Beecham Laboratoires Pharmaceutiques'<br>No holographic label	550<br>Mfg Jun99<br>Exp Jun04<br>{A}	artemisinin$^{\alpha, \beta}$	~70 mg/tablet artemisinin	---------	-----------------------------------
4023 Nigeria<br>6 tablets/blister<br><br>Counterfeit	'Halfan' stated to be manufactured by 'SmithKline Beecham Laboratoires Pharmaceutiques''<br>See 4020	710<br>Mfg May02<br>Exp May05<br><br>{D}	acetaminophen$^{\alpha, \beta}$	~256 mg tablet acetaminophen	---------	-----------------------------------
4024 Nigeria<br>6 tablets/blister<br><br>Counterfeit	'Halfan' stated to be manufactured by 'SmithKline Beecham Laboratoires Pharmaceutiques'<br>Rudimentary 'SB' holographic label (enhancement to label present on 5106)	710<br>Mfg Jun02<br>Exp Jun05<br>{B}	acetaminophen$^{\beta}$	~0.2 mg/tablet acetaminophen	---------	-----------------------------------
5029 Tanzania<br>6 tablets/blister<br><br>Counterfeit	'Halfan' stated to be manufactured by 'SmithKline Beecham Laboratoires Pharmaceutiques'<br>Genuine sample but year of Mfg and Exp altered to extend shelf life by 2 years	666<br>Mfg May03<br>Exp May06	halofantrine hydrochloride$^{\varepsilon}$	250 mg/tablet halofantrine$^{\gamma}$	organic compounds (A), starch (C)	One fungal spore, rare, very small charcoal fragments, pitted plant cells, very common starch granules

5070 Nigeria<br>6 tablets/blister<br><br>Counterfeit	'Halfan' stated to be manufactured by 'SmithKline Beecham Laboratoires Pharmaceutiques'. Same blister design as 5312 with rudimentary (but more sophisticated than 4020 & 4023) 'GSK' holographic label	735<br>Mfg Jan03<br>Exp Jan06	acetaminophen$^{\varepsilon\ \beta}$		talc (A), starch (M), quartz, chlorite and organic compounds	Fungal spores and hyphae, very small charcoal fragments, *Fagopyrum*, *Pinus*, *Artemisia*, Chenopodiaceae, *Sesamum* pollen grains. No wind blown grass pollen suggests seasonally arid area
5106 Cameroon<br>6 tablets/blister<br><br>Counterfeit	'Halfan' stated to be manufactured by 'SmithKline Beecham Laboratoires Pharmaceutiques' Very rudimentary 'SB' holographic label	690<br>Not listed<br>{D}	acetaminophen$^{\alpha,\ \beta}$	302 mg/tablet acetaminophen	---------	-----------------------------------
5176 Democratic Republic of Congo<br>6 tablets/blister<br><br>Counterfeit	'Halfan' stated to be manufactured by 'SmithKline Beecham Laboratoires Pharmaceutiques' Refined version of 'GSK' holographic label with increased clarity in comparison to those on 5070 & 5312 - with finer design elements e.g. micro text (see also 6227, 7103 & 7135E)	768<br>Mfg Jan04<br>Exp Jan07<br>{B}	No API detected$^{\alpha,\ \beta}$	No API detected	---------	----------------------------------

5312 Liberia<br>6 tablets/blister<br><br>Counterfeit	‘Halfan’ stated to be manufactured by ‘SmithKline Beecham Laboratoires Pharmaceutiques’ Same design as 5070 with rudimentary (but more sophisticated than 4020 & 4023)’ GSK’ holographic label	735<br>Mfg Jan03<br>Exp Jan06<br>{E}	acetaminophen$^{\alpha, \beta}$	58.0 mg/tablet acetaminophen	---------	-----------------------------------
6227 China<br>6 tablets/blister<br><br>Counterfeit	‘Halfan’ stated to be manufactured by ‘SmithKline Beecham Laboratoires Pharmaceutiques’ Similar holographic label design to 5176, 7103 & 7135E. Same blister as 7073B apart from changed Mfg and Exp date data and different (enhanced detailed design elements on 7073B) version of ‘GSK’ holographic label	803<br>Mfg Sep04<br>Exp Sep07<br>{C}	acetaminophen$^{\beta}$	< 1mg/tablet acetaminophen	---------	-----------------------------------
7103 Nigeria<br>6 tablets/blister<br><br>Counterfeit	‘Halfan’ stated to be manufactured by ‘SmithKline Beecham Laboratoires Pharmaceutiques’ Similar holographic	781<br>Mfg Jan05<br>Exp Jan08	dipyrone$^{\alpha, \beta}$	------	-----	-----------------------------------

	label design to 5176, 6227 & 7135E					
7073b Nigeria<br>6 tablets/blister<br><br>Counterfeit	'Halfan' stated to be manufactured by 'SmithKline Beecham Laboratoires Pharmaceutiques'. Same blister design as 6227 apart from changed Mfg and Exp date data and different (more rudimentary on 6227) version of 'GSK' holographic label	803<br>Mfg Sep06<br>Exp Sep09	No API detected[ε α]	-------	calcite (A), Starch (C), Talc (M), organic compounds	*Betula* pollen grain and *Stenochlaena* fern spore, fungal spores, very small charcoal fragments, calcite slivers and starch grains, *Pinus* sacci
7135a Nigeria<br>6 tablets/blister<br><br>Genuine	'Halfan' stated to be manufactured by 'SmithKline Beecham Laboratoires Pharmaceutiques'	820<br>Mfg Dec05<br>Exp Dec08	halofantrine hydrochloride[ε]	250 mg/tablet halofantrine hydrochloride[γ]	organic compounds (A), starch (C)	Rare, very small charcoal fragments, pitted plant vessels. No pollen
7135e Nigeria<br>6 tablets/blister<br><br>Counterfeit	'Halfan' stated to be manufactured by 'SmithKline Beecham Laboratoires Pharmaceutiques' Similar holographic label design to 5176, 6227 & 7103	817<br>Exp Dec05<br>Mfg Dec08	pyrimethamine[ε]	-------	talc (A), Starch (M), chlorite	Pollen grains of Cyperaceae and Chenopodiaceae, rare fungal spores, rare, very small charcoal and plant cellular fragments

**D. Counterfeit co-formulated dihydroartemisinin-piperaquine collected in China, destined for Africa. Tablets stated to contain co-formulated dihydroartemisinin-piperaquine (40mg & 320mg). All stated to be manufactured by 'Zheijiang Holley Nanhu Pharmaceutical Group Ltd Under license of HolleyPharm' with stated trade name 'Duo-Cotecxin'**						
China 07/14, Genuine<br>8 blue tablets/blister with packet and leaflet<br>Direct from manufacturer<br><br>Genuine	'Composition per tablet' in English text on packet edge<br>Text transverse across blisterpack<br><br>Hologram	0806<br>09082006<br>08/2008	dihydroartemisinin$^{\alpha, \beta}$, piperaquine$^{\alpha}$	Dihydroartemisinin 38.0, 37.8 mg/tablet, 291, 286 piperaquine mg/tablet	starch (A), quartz (T), clay minerals (T), talc (M)	Fungal spores, black chips, *Pinus*, Poaceae, Moraceae pollen grains, black synthetic thread
China 07/15<br>Genuine<br>8 blue tablets/blister with packet and leaflet<br>Direct from manufacturer<br><br>Genuine		0806<br>09082006<br>08/2008	dihydroartemisinin$^{\alpha, \beta}$, piperaquine$^{\alpha}$	Dihydroartemisinin 37.0, 37.7 mg/tablet, 303, 302 piperaquine mg/tablet	starch (A), clay minerals (T), talc (M)	Rare *Cibotium* fern (Dicksoniaceae) spores, Oleaceae pollen, organic material (~160 μm), numerous black chips
China 07/16<br>Genuine<br>8 blue tablets/blister with packet and leaflet<br>Direct from manufacturer<br><br>Genuine		0806<br>09082006<br>08/2008	dihydroartemisinin$^{\alpha, \beta}$, piperaquine$^{\alpha}$	Dihydroartemisinin 37.3, 37.7 mg/tablet, 292, 299 piperaquine mg/tablet	starch (A), clay minerals (T), talc (M)	Fungal spores, *Cibotium* fern (Dicksoniaceae) spores, *Pinus* pollen, organic debris, numerous black chips
China 07/17<br>Genuine<br>8 blue tablets/blister<br>Direct from manufacturer<br><br>Genuine		0806<br>09082006<br>08/2008	dihydroartemisinin$^{\alpha, \beta}$, piperaquine$^{\alpha}$	Dihydroartemisinin 37.8, 37.0 mg/tablet, 295, 301 piperaquine mg/tablet	starch (A), clay minerals (T), talc (M)	Fungal spores, *Cibotium* fern (Dicksoniaceae) spores, *Pinus* pollen, organic debris, numerous black chips
China 07/18<br>8 blue tablets/blister with packet and leaflet<br><br>Counterfeit	'Composition par tablet', 'Dihydroartemisinine' and Pipéraquine' in English text on packet edge (Fig 1E).	010106<br>11 04 2007<br>04/ 2009	sildenafil$^{\alpha}$	18.4 mg/tablet sildenafil	starch (A), quartz (T), dolomite (M), calcite (T), talc (T)	Abundant fungal spores, rare *Cibotium* fern (Dicksoniaceae) spores, Poaceae pollen grain, organic debris, numerous black chips
China 07/19<br>8 blue tablets/blister with packet and leaflet		010106<br>11 04 2007<br>04/ 2009	sildenafil$^{\alpha}$	7.3 mg/tablet sildenafil	starch (C), quartz (M), dolomite	Abundant fungal spores, hyphae, rare *Cibotium* fern (Dicksoniaceae) spores,

<table>
<tr><td>Counterfeit</td><td rowspan="3">Text less clearly printed and longitudinal across blisterpack
Blisterpack ‘Oral taken’ incorrect English

No hologram

Packet MFD, EXP and LOT codes printed using dot matrix printer</td><td>010106
11 04 2007
04/ 2009</td><td>sildenafil$^{\alpha}$</td><td>7.3 mg/tablet sildenafil</td><td>(C), calcite (M), talc (M)</td><td>Quercus pollen grain, organic debris, charcoal, numerous black chips, fragments of an insect exoskeleton</td></tr>
<tr><td>China 07/20
8 blue tablets/blister with packet and leaflet

Counterfeit</td><td>010106
11 04 2007
04/ 2009</td><td>sildenafil$^{\alpha}$</td><td>9.6 mg/tablet sildenafil</td><td>starch (C), quartz (M), dolomite (C), calcite (M), talc (M)</td><td>Abundant fungal spores, hyphae, Cibotium fern (Dicksoniaceae) spores, Quercus and Chenopodiaceae pollen grains, organic debris, charcoal, numerous black chips, fragments of an insect exoskeleton, mammalian hair</td></tr>
<tr><td>China 07/21
8 blue tablets/blister with packet and leaflet

Counterfeit</td><td>010106
11 04 2007
04/ 2009</td><td>sildenafil$^{\alpha}$</td><td>6.1 mg/tablet sildenafil</td><td>starch (C), quartz (M), dolomite (C), calcite (M), talc (M)</td><td>Abundant fungal spores, hyphae, Cibotium fern (Dicksoniaceae) spores, Quercus, Chenopodiaceae, Pinus, Poaceae, ?Artemisia, ?Euphorbiaceae pollen grains, organic debris, charcoal, numerous black chips, blue and red synthetic threads</td></tr>
<tr><td colspan="7">E. Counterfeit artemether-lumefantrine collected in Ghana and Cameroon. All tablets stated to contain co-formulated artemether-lumefantrine (20mg & 120mg)</td></tr>
<tr><td>Gh 09/01
8 yellow tablets/blister with packet and leaflet

Counterfeit</td><td>‘Coartem’ stated to be manufactured by ‘Beijing Novartis Pharma Ltd, Beijing, China for Novartis Pharma AG, Basle, Switzerland, under licence from the PRC.’ Packet text in</td><td>M1200
01 2009
05 2011</td><td>Pyrimethamine
Yellow pigment of unknown identity</td><td>Pyrimethamine 6.2 & 7.7 mg/tablet
Yellow pigment of unknown identity</td><td>starch (A)</td><td>Remained yellow during acetolysis. Rare fungal hyphae and spores, Asteraceae and Poaceae pollen, other broken unidentifiable pollen</td></tr>
</table>

	English, French, German and Spanish. German spelling error ‘Unter 30°C lagem’. No blisterpack codes. Blisterpack corners angled					
Gh 09/02<br>8 yellow tablets/blister with packet and leaflet<br><br>Counterfeit	‘Coartem’ stated to be manufactured by ‘Beijing Novartis Pharma Ltd, Beijing, China for Novartis Pharma AG, Basle, Switzerland, under licence from the PRC.’ Packet text in English, French, German and Spanish. Differences in packet size and colour. Text in different font. Correct German phrase ‘‘Unter 30°C lagern’. Different widths and colours of packet colour tab bars, Blisterpack size different,	X0089<br>07 2008<br>08 2010	Pyrimethamine<br>Yellow pigment of unknown identity	Pyrimethamine 24.0 & 25.0 mg/tablet<br>Yellow pigment of unknown identity	talc (A), starch (M)	Remained yellow during acetolysis. Large quantity of starch grains that did not dissolve. Rare fungal remains. A few *Pinus* pollen grains

	codes smudged, foil duller and corners curved. Leaflet size different and paper colour different					
CAM 10/01<br><br>Cameroon, from Novartis via INTERPOL<br><br>6 yellow tablets/blister with packet and leaflet<br><br>Counterfeit	'Coartem' stated to be manufactured by 'Beijing Novartis Pharma Ltd, Beijing, China for Novartis Pharma AG, Basle, Switzerland, under licence from the PRC.' Packet text in English, French, German and Spanish.<br><br>NAFDAC Reg. No. 04-3275 Blisterpack corners angled	X1257<br>06 2008<br>05 2011<br><br>Indistinct printing on blister	Pyrimethamine and sulfadiazine	No artemether or lumefantrine<br><br>Pyrimethamine 148 & 139 mg/tab	starch (A)	A lot of very fine organic and non-organic debris. The organic debris consists of very finely disarticulated plant material, black specks and rare fragments of charcoal. The only spores and pollen are one *Pinus* grain and two Chenopodiaceae grains (*Chenopodium*?). Suggests an arid climate. The combination of *Pinus* plus chenopod pollen on their own are consistent with, but do not prove, a source in India. Fungal spores very common and varied in morphology - single celled, multicellar, clumps and variously ornamented
CAM 10/02<br><br>Cameroon, from Novartis via INTERPOL<br><br>6 yellow	'Coartem' stated to be manufactured by 'Beijing Novartis Pharma Ltd, Beijing, China for Novartis Pharma AG, Basle,	X1257<br>06 2008<br>05 2011<br><br>Indistinct printing on blister	Pyrimethamine and sulfadiazine	No artemether or lumefantrine<br><br>Pyrimethamine 148 & 145 mg/tablet	starch (A)	Identical to CAM 10/01 except contains much less finely dispersed organic and inorganic material and no chenopod pollen. One *Pinus* grain is present. Fungal spores and hyphae rare.

tablets/blister with packet and leaflet<br><br>Counterfeit	Switzerland, under licence from the PRC.' Packet text in English, French, German and Spanish. NAFDAC Reg. No. 04-3275 Blisterpack corners angled					
Ken 06/01<br>From Novartis agent in Kenya<br>8 yellow tablets/blister with packet and leaflet<br><br>Genuine	'Coartem' stated to be manufactured by 'Beijing Novartis Pharma Ltd, Beijing, China for Novartis Pharma AG, Basle, Switzerland, under licence from the PRC.' Packet text in English, French, German and Spanish	X0124<br>08 2005<br>07 2007	Artemether, lumefantrine	Artemether 35.0 & 35.0 mg/tab and lumefantrine 314 & 317 mg/tablet	starch (C), organic compounds (C), calcium sulphate (C), anhydrite (M), talc (T)	Turned dark brown during acetolysis. Very little seen, apart from some fragmentary conifer pollen grains and rare fungal remains. Similar to Lao 09/06
NOV 10/43 Direct from manufacturer. 6 tablets/blister with packet and leaflet<br><br>Genuine	'Riamet' stated to be manufactured by 'Beijing Novartis Pharmaceutical Ltd.'	X0095<br>09 2007<br>08 2009	Artemether, lumefantrine	Artemether 19.3, 19.5, 19.5 mg/tablet & 120, 117, 117 lumefantrine mg/tablet	------	----------

NOV 10/44. Direct from manufacturer. 6 tablets/blister with packet and leaflet<br><br>Genuine	'Riamet' stated to be manufactured by 'Beijing Novartis Pharmaceutical Ltd.'	X0102<br>11 2008<br>10 2010	Artemether, lumefantrine	Artemether 19.3, 19.4, 19.3 mg/tablet & 119, 120, 120 lumefantrine mg/tablet	------	----------
NOV 10/45. Direct from manufacturer. 6 tablets/blister with packet and leaflet<br><br>Genuine	'Riamet' stated to be manufactured by 'Beijing Novartis Pharmaceutical Ltd.'	X0106<br>11 2009<br>10 2011	Artemether, lumefantrine	Artemether 19.4, 19.6, 19.3 mg/tablet & 119, 120, 119 lumefantrine mg/tablet	------	----------
Lao 09/06, 09/07 & 09/09. From CMPE stock, Government of Laos sourced from Novartis 24 yellow tablets/blister<br><br>Genuine	'Coartem' stated as manufactured by 'Novartis Pharmaceutical Coroporation, Suffern, New York, USA for Novartis Pharma AG, Basle, Switzerland, under licence.' Text in English	F1350<br>02 2009<br>01 2011	Artemether, lumefantrine for Lao 09/09	Artemether 45.2 & 44 mg/tablet and 287 & 280 lumefantrine mg/tablet for Lao 09/09	starch (C), organic compounds (C), calcium sulphate (C), anhydrite (M), talc (T)	Lao 09/06 and Lao 09/07. Turned dark brown during acetolysis. Rare starch grains, very rare fungal hyphae. Conifer pollen, including *Pinus* and *Dacrydium pierrei*, Cyatheaceae and smooth unidentifiable monolete spores & pollen of *Betula*, *Trifolium*, *Alnus, Carpinus*, ?*Quercus*, Proteaceae, Rubiaceae (coffee family) & unidentifiable tricolporate grains
**F. Convenience sampling of antimalarials in the Democratic Republic of the Congo (DRC)**						
DRC 08/01. Central market Kananga, Lulua Province, DRC. 6x100mg white artesunate tablets/blister with packet<br><br>Poor quality	'Marinate' No manufacturers details on the packet or blister. Only GUJ/DRUGS/1407 *	RET. 008<br>JAN.2006<br>DEC.2008	artesunate	artesunate 79.0, 88.0 mg/tablet	----	----

DRC 08/02. Central market Kananga, Lulua Province, DRC. 12x50mg white artesunate tablets/blister with packet<br><br>Genuine	'Artesunate' Stated to be manufactured by 'Mekophar Chemical Pharmaceutical Joint-Stock Company, HCMC Vietnam.' 'Fabrique pour "AMT Inc. USA"<br>Text on packet in English, Spanish and French	07011FX<br>03/2007<br>03/2010	artesunate	artesunate 45.0, 52.5, 53.5, 52.5 mg/tablet	-----	-----
DRC 08/03. Central market Kananga, Lulua Province, DRC. 12x50mg white artesunate tablets/blister with packet<br><br>Genuine	'Artesunat' Stated to be manufactured by 'Mekophar Chemical Pharmaceutical Joint-Stock Company, HCMC Vietnam.' 'Fabrique pour "AMT Inc. USA"<br>Text on packet in English, Spanish and French	07013FX<br>03/2007<br>03/2010	artesunate	artesunate 49.0, 49.0, 55.0, 53.0 mg/tablet	-----	-----
DRC 08/04. Central market Kananga, Lulua Province, DRC. 6x100mg white artesunate tablet/blister with packet<br><br>Genuine	'ARTSUN-100' Stated to be 'Made for euromedi Pharma 99, Carmelite Road, Middlesex, London'<br>Text in English and French<br>Code No. MP/DRUGS/25/23/2001 *	AK-02<br>FEB-07<br>JAN-10	artesunate	artesunate 80.0, 90mg/tablet	-----	-----

DRC 08/05. Central market Kananga, Lulua Province, DRC.<br>12x50mg white artesunate tablet/blister with packet<br><br>Genuine	'Sunat'<br>Stated to be manufactured by 'SHALINA LABORATORIES PVT. LTD. 96, Maker Chamber VI, Nariman Point, Mumbai-Inde'<br>Code No. MH/DRUGS/KD-1392A *	006<br>NOV.06<br>SEP.09	artesunate	artesunate 45.0, 50.0, 54.0, 52.0 mg/tablet	-----	-----
DRC 08/06. Central market Kananga, Lulua Province, DRC.<br>3x306.2mg amodiaquine tablets and 3x100mg white artesunate tablets/blister with packet<br><br>Genuine	'Sunat-A'<br>Stated to be manufactured by 'SHALINA LABORATORIES PVT. LTD. 96, Maker Chamber VI, Nariman Point, Mumbai-Inde'<br>Code No. MH/DRUGS/KD-1392A *	002<br>OCT.07<br>SEP.10	1. amodiaquine $^{\alpha}$<br>2. artesunate, disaccharide$^{\alpha, \beta}$	1. Amodiaquine 242, 288 mg/tablet<br>2. Artesunate 87.0, 101 mg/tablet	-----	-----
DRC 08/07. Central market Kananga, Lulua Province, DRC.<br>12x50mg white artesunate tablets/blister with packet<br><br>Genuine	'Artesunat' Stated to be manufactured by 'Mekophar Chemical Pharmaceutical Joint-Stock Company, HCMC Vietnam.'<br>'Fabrique pour "AMT Inc. USA"<br>Text on packet in English, Spanish and French	07014FX<br>03/2007<br>03/2010	artesunate acid$^{\beta}$	artesunate 45.0, 54.0, 54.0, 52.0 mg/tablet	-----	-----

DRC 06/01, Kivu, DRC. 1 30ml bottle of suspension with spoon, packet and leaflet<br><br>Counterfeit	'HALFAN' Suspension Stated to be manufactured by 'SmithKline Beecham Laboratoire Pharmaceutiques Esplanade Charles de Gaulle 92731 NANTERRE Cedex,'<br><br>NAFDAC REG NO. 04-2181 *	BN 3006<br>Mfg 07/2003<br>Exp 07/2006	No API detected	---------	--------	Abundant extremely fine black material, less than 0.25µm in diameter. Rare fungal remains, all of the same type, and 8 pollen grains including 5 small *Pinus* grains, and one Poaceae
DRC 07/01, Kinshasa, DRC. 6 x100mg artesunate tablets/blister<br><br>Counterfeit	Astrinate on one face of leaflet and Arinate on reverse<br>Stated to be 'Manufactured for AT17 RUE POISSONNIERS 75018 PARIS' *	Lot: 110705<br>Exp: 06/08<br>Mfg: 07/05	artesunate	88.5, 70.9 mg artesunate/tablet	----	-----
**G. Convenience sampling of antimalarials in West Africa**						
BF 08/01<br>8 x coformulated dihydroartemisinin-piperaquine (40mg & 320mg) tablets/blister with packet<br>Pharmacy, Ouagadougou, Burkina Faso<br><br>Genuine	'DUO-COTECXIN'<br>Stated to be manufactured by 'Zheijiang Holley Nanhu Pharmaceutical Group Co. Ltd. Under license of HolleyPharm'	020207<br>270022007<br>02/2009	dihydroartemisinin$^{\beta}$, piperaquine$^{\beta}$	Dihydroartemisinin 53.0, 32.0 mg/tablet, piperaquine 240 mg/tablet	-----	-----
BF 08/02<br>6 x 100mg artesunate tablet/blister with packet	'Arinate'<br>Stated to be manufactured by 'Dafra Pharma nv / sa	07C261<br>26 Mar 2007<br>03 2010	artesunate$^{\beta}$	artesunate 98.0, 106 mg/tablet	-----	-----

Pharmacy, Ouagadougou, Burkina Faso<br><br>Genuine	Turnhout (Belgium)' *					
BF 08/03<br>6x50mg artesunate tablets/blister<br>Pharmacy, Ouagadougou, Burkina Faso<br><br>Genuine	'Artesunate' Stated to be manufactured by 'Ubithera, Adam Pharm Comp Ltd, Anhui, China' *	B. N.: 030906<br>MFG: 09/06<br>EXP: 08/09	artesunate$^{\beta}$	artesunate 44.0, 50.0 mg/tablet	-----	-----
TOG 08/01<br>12x50mg artesunate tablets/blister with packet<br>Pharmacy, Lome, Togo<br><br>Genuine	'Artesunate' Stated to be manufactured by 'TONGMEI LABORATOIRE, Lomé-TOGO' *	010307<br>03 2007<br>03 2010	artesunate$^{\beta}$	artesunate 44.0, 51.0, 50.0, 48.0 mg/tablet	-----	-----
TOG 08/02<br>12x50mg artesunate tablets/blister with packet<br>Pharmacy, Lome, Togo<br><br>Genuine	ARTESUNATE' Stated to be from 'Arthésis Nyd Pharma, Genéve-Suisee. Manufactured by Laboratoires Cipla Ltd MH/DRUGS/845' *	K60703<br><br>07/2008	artesunate$^{\beta}$	artesunate 50.0, 53.0, 55.0, 50.0 mg/tablet	-----	-----
BEN 08/01<br>12x50mg artesunate tablets/blister with packet<br>Market, Cotonou, Benin<br><br>Genuine	'ARTESUNATE' Stated to be manufactured by 'NSW Pharmaceutical Co. Ltd. Under the License of Imperial Botanical CJS INT'L.' Company logo on packet similar design to Guilin Pharmaceutical Co. Ltd hologram but with 'IMPERIAL BOTANICAL' across the mountains *	070522<br>05 / 2007<br>04 / 2010	artesunate$^{\beta}$	artesunate 43.0, 47.0, 46.0, 46.0 mg/tablet	-----	-----

BEN 08/02<br>3x200mg white artesunate tablets and 3x pink tablets of 500mg sulfamethoxypyrazine and 25mg pyrimethamine/ blister with packet<br>Pharmacy, Cotonou, Benin<br><br>Genuine	'AsunateDenk 200 Plus' Stated to be manufactured by<br>'Artesan Pharma GmbH & Co. KG, Lüchow, Germany for DENK PHARMA GmbH & Co. KG, Muenchen, Germany'<br>'Reg. No. NAFDAC…,ZAM 130/032-POM'	14861<br>08 / 2006<br>08 / 2009	1. artesunate$^{\alpha, \beta}$<br>2. sulfamethoxypyrazine$^{\alpha}$, pyrimethamine$^{\alpha, \beta}$	1. Artesunate 182, 230 mg/tablet<br>2. sulfamethoxypyrazine 495, 501, 718 mg/tablet, pyrimethamine 9.3, 30.0, 36.0 mg/tablet	-----	-----
BEN 08/03<br>6 coformulated tablets of 100mg artesunate and amodiaquine 270mg (base)/blister with packet<br>Pharmacy, Cotonou, Benin<br><br>Genuine	'Coarsucam' Stated to be manufactured for 'sanofi Aventis, Casablanca, Morocco, manufactured by Maphar Casablanca, Morocco'	5010<br>06 / 2007<br>06 / 2009	artesunate$^{\alpha, \beta}$, amodiaquine$^{\alpha}$	artesunate 105, 99.0 and amodiaquine 227, 265 mg/tablet	-----	-----
BEN 08/04<br>8x 60mg dihydroartemisinin tablets/blister with packet<br>Pharmacy, Cotonou, Benin<br><br>Genuine	'ARTEMAX' Stated to be manufactured by 'G.A. Pharmaceuticals S.A. Athens, Greece' *	6060<br>1 2008<br>1 2010	dihydroartemisinin$^{\beta}$	dihydroartemisinin 65.0, 28.0 mg/tablet	-----	-----
BEN 08/05<br>3x4 x 50mg artesunate tablets, 4 tablets/ blister, three blisters with packet<br>Pharmacy, Cotonou, Benin<br><br>Genuine	'Artemal-S' Stated to be manufactured by<br>'Plethico Pharmaceuticals Limited<br>Indore (MP), India<br>ML. 25/39/85' *	7450<br>06/2007<br>05/2010	artesunate$^{\beta}$	artesunate 46.8mg/tablet	-----	-----

GH 08/01<br>12x 50mg artesunate tablets/blister with packet<br>Pharmacy, Tamale, Ghana<br><br>Genuine	MALASATE 50'' Stated to be manufactured by 'ERNEST CHEMISTS LIMITED, Accra, Ghana' *	0810G<br>10-07<br>10-10	artesunate[β]	artesunate 50.0, 47.0, 50.0, 47.0 mg/tablet	-----	-----
GH 08/02<br>3x4 x 50mg artesunate tablets, 4 tablets/blister, three blisterpacks with packet<br>Pharmacy, Tamale, Ghana<br><br>Genuine	'ARTESUNATE XL'<br>Stated to be manufactured by 'XL LABORATORIES PVT. LTD, Bhiwadi, Rajasthan, India for KAMA INDUSTRIES LTD. ACCRA-NORTH, GHANA'<br>Mfg. Lic. No. : Raj. 2059 *	7002<br>05/2007<br>05/2010	artesunate[β]	artesunate 45.0, 48.5, 52.5, 50.0 mg/tablet	-----	-----
GH 08/03<br>12x50mg artesunate and 12x 150mg amodiaquine (base) tablets/blister<br>Pharmacy, Navrongo, Ghana<br><br>Genuine	'MALATEX'<br>Stated to be manufactured by 'KINAPHARMA LIMITED<br>Accra, Ghana' *	B • N001<br><br>02/11	1. artesunate[α, β]<br>2. amodiaquine[α]	1. artesunate 47.0, 53.0, 53.0, 54.0 mg/tablet<br><br>2. amodiaquine 151, 140, 144, 152 mg/tablet	-----	-----
GH 08/04<br>6x100mg artesunate and 6x 300mg amodiaquine (base) tablets/blister with packet<br>Pharmacy, Bolgatanga, Ghana<br><br>Genuine	'GSUANTE 100 KIT'<br>Stated to be manufactured by 'GVS LABS Dombivili, India'<br>Mfg. Lic. No.: KD-1562 A	G0-01<br>16/01/2006<br>15/01/2009	1. artesunate[α, β]<br>2. amodiaquine[α]	1. artesunate 81.0, 90.0 mg/tablet<br>2. amodiaquine 260, 246 mg/tablet	-----	-----

GH 08/05<br>3x50mg artesunate powder sachets and 3x150mg amodiaquine powder sachets in packet<br>Pharmacy, Bolgatanga, Ghana<br><br>Genuine	'CAMOSUNATE PED'<br>Stated to be manufactured by 'DANPONG-ADAMS PHARMACEUTICAL INDUSTRY (GHANA) LIMITED ACCRA, GHANA' *	0702006<br>02/07<br>02/10	1. artesunate$^{\beta}$<br>2. amodiaquine$^{\beta}$	1. artesunate 52.0 mg/tablet<br>2. amodiaquine 131 mg/tablet	-----	-----
GH 08/06<br>6x200mg artesunate tablets/blister with packet<br>Pharmacy, Kumasi, Ghana<br><br>Genuine	'ARTENEX 200'<br>Stated to be manufactured by 'KINAPHARMA LIMITED, Accra, Ghana' *	007<br>SEP 05<br>SEP 09	artesunate$^{\beta}$	artesunate 173, 212 mg/tablet	-----	-----
GH 08/07<br>8x60mg dihydroartemisinin tablets/blister with packet<br>Pharmacy, Kumasi, Ghana<br><br>Genuine	'COTECXIN'. Stated to be manufactured by 'Jiaxing Nanhu Pharmaceutical Co. Ltd. Jiaxing City, under license of Beijing Holley-Cotec Pharmaceuticals'	010106<br>19012006<br>01/2009	dihydroartemisinin$^{\beta}$	dihydroartemisinin 58.0, 13.0 mg/tablet	-----	-----
GH 08/08<br>12x50mg artesunate tablets/blister with packet<br>Pharmacy, Kumasi, Ghana<br><br>Genuine	'ACUMAL'<br>Stated to be manufactured by 'JCPL Pharma Pvt. Ltd, Jalgaon for KOJACH LIMITED KUMASI-GHANA'<br>Mfg. Lic. No. 514<br>For Batch No, Mfd and exp. Date packet states 'See on Strip' *	B/No.ET 504<br>DEC.2005<br>NOV.2009	artesunate$^{\beta}$	artesunate 44.0, 45.0, 45.0, 46.0 mg/tablet	-----	-----

GH 08/09<br>8x60mg dihydroartemisinin tablets/blister with packet<br>Pharmacy, Akisombo, Ghana<br><br>Genuine	'ALAXIN'<br>Stated to be manufactured by 'GVS LABS, Dombivili, INDIA'<br>Mgf. Lic. : KD 1562 A	GO – 26<br>02/01/2007<br>01/01/2010	dihydroartemisinin$^{\beta}$	dihydroartemisinin 63.0, 37.0 mg/tablet	-----	-----
GH 08/10<br>12x50mg artesunate tablets/blister with packet<br>Pharmacy, Akisombo, Ghana<br><br>Genuine	'ARTESUNATE'<br>Stated to be manufactured by 'Guilin Pharmaceutical Co. Ltd., Guilin, Guangxi, China'	070506<br>05/2007<br>05/2010	artesunate$^{\beta}$	artesunate 49.0, 50.0, 52.0, 53.0 mg/tablet	-----	-----
GH 08/11<br>6x100mg artesunate tablets/blister with packet<br>Pharmacy, Hohoe, Ghana<br><br>Genuine	'GSUNATE FORTE'<br>Stated to be manufactured by 'BLISS GVS PHARMA LTD. Palghar, Maharasthra, INDIA'<br>Marketed by: 'TOBINCO PHARMACEUTICAS LTD. Accra, Ghana'<br>Mfg. Lic. No. : 1040	GE – 08<br>08 / 2007<br>07 / 2010	artesunate$^{\beta}$	artesunate 88.0, 100 mg/tablet	-----	-----
GH 08/12<br>12x50mg white artesunate tablets and 12x153.1mg amodiaquine (base) yellow tablets/blister with packet<br>Pharmacy, Hohoe, Ghana<br><br>Genuine	'GSUNATE 24 KIT'<br>Stated to be manufactured by 'BLISS GVS PHARMA LTD. Palghar, Maharasthra, INDIA'<br>Mfg. Lic. No. : 1040	KF – 05<br>12 / 2007<br>11 / 2010	1. artesunate$^{\beta}$<br>2. amodiaquine$^{\beta}$	1. artesunate 42.0, 48.0, 51.0, 52.0 mg/tablet<br>2. amodiaquine 137, 156, 162, 156 mg/tablet	-----	-----

GH 08/13<br>6 Artesunate100mg tablets and 6<br>amodiaquine 300mg tablets/blister with packet<br>Pharmacy, Ho, Ghana<br><br>Poor quality	'AMONATE-400'<br>Stated to be manufactured for 'Pharmanova Limited, Accra, Ghana.<br>Manufactured by: Atlantic Pharmaceuticals Limited Accra, Ghana' *	7004<br>10-07<br>10-10	1. artesunate[α, β]<br>2. amodiaquine[α]	1. artesunate 92.0, 103 mg/tablet<br>2. amodiaquine 237, 240 mg/tablet	-----	-----
GH 08/14<br>co-formulated 8 tablets of dihydroartemisinin 40mg and piperaquine 320mg/ blister with packet<br>Pharmacy, Aflao, Ghana<br><br>Genuine	'P-ALAXIN'<br>Stated to be manufactured by 'GVC LABS, Dombivili, INDIA'<br>Mfg Lic.: KD-1485A<br>Blisterpack M•I•KD•1485A	Packet<br>Batch No.: GB-01<br>Mfg.Date : 08/ 2005<br>Exp. Date : 07/ 2008<br><br>Blister<br>GB•01.08/05. 07/08	dihydroartemisinin, piperaquine, disaccharide	dihydroartemisinin 49.0, 21.0 mg/tablet, piperaquine 243 mg/tablet	-----	-----
GH 08/15<br>12x50mg artesunate tablets/blister with packet<br>Pharmacy, Bremen-Asikuma, Ghana<br><br>Poor quality	'LEVER Artesunate'<br>Stated to be manufactured by<br>'ADAMS PHARAMCEUTICAL (ANHUI) CO., LTD. ANHUI, CHINA<br>Division of Sunflower Int'l Group'<br>NAFDAC REG. NO.: 04-5865<br>Marketed by GENEITH GLOBAL LIMITED *	040207<br>02/07<br>01/10	artesunate[β]	artesunate 39.0, 43.0, 47.0, 47.0 mg/tablet	-----	-----

GH 08/16<br>3x50mg artesunate and 3x153mg amodiaquine (base) tablets /blister with packet<br>Pharmacy, Accra, Ghana<br><br>Genuine	'ARSUCAM'<br>Stated to be manufactured by 'Sanofi~synathelabo Manufacturer MAPHAR – Casablanca –Morocco'	0244<br>02/06<br>02/08	1. artesunate[β]<br>2. amodiaquine[β]	1. artesunate 47.0, 55.0 mg/tablet<br>2. amodiaquine 134, 144 mg/tablet	-----	-----

[*] Genuine sample from manufacturer not available for comparison [α]Active Pharmaceutical Ingredient (API) detected by DESI-MS, [β]API verified by DART-MS, [γ] GSK tablets are manufactured to 250mg of halofantrine hydrochloride and this was not verified by HPLC in this work, [ε]API detected by Fourier Transform Infra Red spectroscopy (FTIR) and verified when necessary by high performance liquid chromatography with electrospray ionisation and mass spectrometry detection (HPLC-ESI-MS)

A (abundant, > 60%), C (common, 20-60%), M (minor, 5-20%), T (trace < 5%). Clay minerals are probably smectite and chlorite.

HPLC-UV = high performance liquid chromatography with ultraviolet detection, XRD = X-ray diffraction, GSK = GlaxoSmithKline, SB = SmithKline Beecham, LSHTM – HPLC analysis performed by HK at London School of Hygiene and Tropical Medicine – see Ioset & Kaur (2009), IN = insufficient material left for analysis

[1] Reported in Ioset & Kaur (2009)

Meta-alunogen is aluminium sulphate hydrate
Acetaminophen is paracetamol
