# Supplementary material for: Poor quality vital anti-malarials in Africa - an urgent neglected public health priority
Source: Malar J. 2011 Dec 13;10:352. doi: 10.1186/1475-2875-10-352 (PMC3262771; doi:10.1186/1475-2875-10-352)
Supplement: Additional file 3 — Features of counterfeit artesunate labelled as made by Mekophar Chemical Pharmaceutical Joint-Stock Company. Distinguishing features in red font. [file 1475-2875-10-352-S3.PDF]

Additional File 3. Features of counterfeit artesunate labelled as made by 'Mekophar Chemical Pharmaceutical Joint-Stock Company'. Distinguishing features in red font.

Variable	Counterfeit		Genuine
**Packet**	**Cameroon S5/07**	**Cameroon S5/08**	**Mek 10/03 & Mek 10/05**
Colour of inside surface	Cream	Cream	White
Packet white area RGB %	99.6/98.1/99.5	97.1/95.2/95.8 *	96.5/96.8/99.3
Packet light green area RGB %	68.3/88.5/59.0	68.8/87.8/65.3 *	75.8/90.7/73.6
Packet dark green area RGB %	11.9/57.0/10.1	13.3/59.5/12.1 *	22.1/65.3/20.4
Packet height at tallest point/cm	15.5	15.4 – 15.6	15.4
Packet max width when open/cm	9.9	9.7 - 9.8	10.0
Hologram	'MKP'	'MKP'	'NEROS-MEKOPHAR' & 'NPL NEROS' in centre
Bar Code	8 934574010511	8 934574010511	8 934574010511
Packet weight/g Median (range)	3.96	3.94 (3.85-4.07) *	3.57 (3.55-3.58)
Text language	English & French	English & French	English & French
	Manufactured by Mekophar Chemical Pharmaceutical Joint-Stock Company 297/5 Ly Thuong Kiet St,-Dist, 11-Ho Chi Minh City-Vietnam	Manufactured by Mekophar Chemical Pharmaceutical Joint-Stock Company 297/5 Ly Thuong Kiet St,-Dist, 11-Ho Chi Minh City-Vietnam	Manufactured by Mekophar Chemical Pharmaceutical Joint-Stock Company 297/5 Ly Thuong Kiet St,-Dist, 11-Ho Chi Minh City-Vietnam
REG. N^{O}	VNA-2892-99	VNA-2892-99	VNB – 2339-04
NAFDAC REG. N^{o}	04-3397	04-3397	04-3397
LOT	0170805	06027FX	10005FX
EXP	08/ 2005	07/ 2006	25/03/10
MFD	08/ 2008	07/ 2009	25/03/13
	Marketed by: NEGROS PHARMACEUTICALS LTD., LAGOS NIGERIA	Marketed by: NEGROS PHARMACEUTICALS LTD., LAGOS NIGERIA	Marketed by: NEGROS PHARMACEUTICALS LTD., LAGOS NIGERIA
**Blisterpack**			
Tablets per blisterpack	12	12	12
Hologram	no	no	no
Batch number, Mfg Date, Expiry Date		06027FX 07-2009	10005FX 250313
Details embossed or printed		Embossed	Embossed

**Leaflet**			
Dimensions/cm	10.6 x 15.9	10.4-10.6 x 15.9	10.4 -15.6
Text language	English & French	English & French	English & French
Paper colour	Matt white	Matt white	Matt white
Blank paper RGB %	93.6/90.4/92.1	97.9/96.7/96.0	96.0/96.8/99.4
