# Supplementary material for: Poor quality vital anti-malarials in Africa - an urgent neglected public health priority
Source: Malar J. 2011 Dec 13;10:352. doi: 10.1186/1475-2875-10-352 (PMC3262771; doi:10.1186/1475-2875-10-352)
Supplement: Additional file 4 — Distinguishing features of counterfeit DHA labelled as made by Jiaxing Nanhu Pharmaceutical Co. Distinguishing features in red font. [file 1475-2875-10-352-S4.PDF]

Additional file 4. Distinguishing features of counterfeit DHA labelled as made by 'Jiaxing Nanhu Pharmaceutical Co.'. Distinguishing features in red font.

**Variable**	**Genuine**	**Counterfeit**
**Packet**	**Kenya 07/01**	**Kenya 07/02**
Colour of inside surface	Matt white	Matt white
White area on packet %RBG	96.1/96.7/97.6	5.4/59.6/39.7
Green symbol on packet RBG %	97.2/95.6/94.8	11.8/59.9/40.5
Packet weight/g	4.80	4.74
Packet height at tallest point/cm	15.4	15.3
Packet max width when open/cm	10.3	10.2
Text language	English & French	English & French
	Jiaxing Nanhu Pharmaceutical Co., Ltd. No. 205, Yunhai Road, Economy Development Zone, Jiaxing City<br>Mfg under license of: Beijing Holley-Cotec Pharmaceuticals Co., Ltd.	Jiaxing Nanhu Pharmaceutical Co., Ltd. No. 205, Yunhai Road, Economy Development Zone, Jiaxing City<br>Mfg under license of: Beijing Holley-Cotec Pharmaceuticals Co., Ltd.
LOT	031106	030406
EXP	11/2009	04/2009
MFD	10112006	10042006
	Printed	Printed
**Blisterpack**		
No. tablets	8	8
Tablet	White, Ro symbol with 'R' upside down	White, Ro symbol with 'R' upside down
Tablet diameter/thickness/mm	9.22/3.98	9.16/5.59
Tablet colour RBG %	80/75/73	56/51/43
Blister diameter/mm	10	11
Blister foil on tablet side	Dark orange	Light orange
Blister foil on tablet side RBG%	68.2/32.0/6.5	57.1/39.0/17.5
Batch number, Expiry Date	LOT 031106<br>EXP11/2009	LOT030605<br>EXP04/2009
	Embossed	Embossed
**Leaflet**		
Dimensions/cm	21.0 x 14.7	21.0 x 14.8
Paper colour	Matt	Matt
Blank paper RGB %	93.3/93.3/96.4	93.1/92.0/94.4
Text language	English & French	English & French
