# Supplementary material for: Poor quality vital anti-malarials in Africa - an urgent neglected public health priority
Source: Malar J. 2011 Dec 13;10:352. doi: 10.1186/1475-2875-10-352 (PMC3262771; doi:10.1186/1475-2875-10-352)
Supplement: Additional file 5 — Distinguishing features of counterfeit DHA-piperaquine labelled as made by Zheijiang Holley Nanhu Pharmaceutical Co., Ltd. Distinguishing features in red font. * medians, + medians (range). [file 1475-2875-10-352-S5.PDF]

Additional file 5. Distinguishing features of counterfeit DHA-piperaquine labelled as made by 'Zheijiang Holley Nanhu Pharmaceutical Co., Ltd.'. Distinguishing features in red font. * medians, + medians (range)

**Variable**	**Genuine**	**Counterfeit**
**Packet**	**China 07/14-17**	**China 07/18-21**
Colour of inside surface	Matt white	Creamy white
Blue area on packet RBG %	5.5/33.6/80.8 *	8.4/34.6/80.0 *
White area on packet RBG %	94.5/94.4/94.0 *	94.7/94.3/93.2 *
Packet label	DUO-COTECXIN® Manufactured by Zheijiang Holley Nanhu Pharmaceutical Co., Ltd. 205, Yunhai Road, Economy Development Zone, Jiaxing City, P.R. China Under license of Holleypharm	DUO-COTECXIN® Manufactured by Jiaxing Nanhu Pharmaceutical Co., Ltd. No. 205, Yunhai Road, Economy Development Zone, Jiaxing City Under license of Holleypharm
Hologram	'FACT DUO-COTECXIN'	No
Packet weight/g (median (range))	6.84 (6.77-6.95) +	7.79 (7.76 – 7.84) +
Packet height at tallest point/cm	17.5	17.5 – 17.6
Packet max width when open/cm	13	12.9
Text language	English and French	English and French
LOT	040806	010106
EXP	08/2008	04/ 2009
MFD	09082006	11 04 2007
		Different font
Dosage on side	Correct English and French text	Franglais in English section 'Composition par tablet' 'Dihydroartemisinine' 'Pipéraquine'
**Blisterpack**		
No tablets	8	8
Tablet diameter/thickeness mm	12.21/4.84	12.14/5.72
Codes	EXP08/2008 LOT040806	EXP04/2009 LOT010106
Tablet	Blue coated, scored and on obverse 'DO' White inside	Blue coated, scored and on obverse 'DO' White inside
Tablet colour	51.0/67.8/94.5 *	48.2/61.8/90.9 *
**Leaflet**		
Dimensions/cm	21 x 14.5	20.8 – 20.9 x 14.5 - 14.6
Paper colour	Matt white	Matt white
Blank paper RGB %	95.2/94.8/94.0	93.4/93.0/92.3
Text language	English and French	English and French
