# Supplementary material for: Poor quality vital anti-malarials in Africa - an urgent neglected public health priority
Source: Malar J. 2011 Dec 13;10:352. doi: 10.1186/1475-2875-10-352 (PMC3262771; doi:10.1186/1475-2875-10-352)
Supplement: Additional file 6 — Distinguishing features of counterfeit artemether-lumefantrine labelled as manufactured by Beijing Novartis Pharma Ltd, Beijing, China for Novartis Pharma AG, Basle, Switzerland. Distinguishing features in red font. [file 1475-2875-10-352-S6.PDF]

Additional file 6. Distinguishing features of counterfeit artemether-lumefantrine labelled as manufactured by 'Beijing Novartis Pharma Ltd, Beijing, China for Novartis Pharma AG, Basle, Switzerland'. Distinguishing features in red font.

**Variable**	**Counterfeit**		**Genuine**
**Packet**	**Gh 09-01**	**Gh 09-02**	**Ken 06-01**
Colour of inside surface	grey	whiter	greyer
Inside surface RGB %	82.6/77.2/74.9	99.5/97.1/96.0	97.9/95.3/96.0
White area RGB %	95.4/93.1/90.5	97.1/94.0/92.0	97.2/93.5/93.2
Yellow area RGB %	95.7/70.1/1.8	95.2/73.7/13.9	100.0/71.3/7.5
Red text '20/120' RGB %	97.5/25.5/25.4	88.9/25.3/33.0	99.0/27.4/30.0
Top box side height/mm	20.0	20.0	20.0
Middle Right tab width/mm	33.5	31.5	31.5
Top Right tab flap width/mm	16.5	17.5	16.5
Bottom Right flap width/mm	16.5	18.5	16.5
Packet height at tallest point/mm	142.0	143.5	143.0
Packet max width when folded/mm	104.0	104.0	105.0
Packet Top left tab width/mm	16.5	17.5	16.5
Middle Left tab width/mm	18.0	18.0	17.0
Bottom Left flap width/mm	33.5	31.5	32.0
Packet weight/g	4.87	5.13	5.14
Text	Less clear<br>Accents of different font	Clear<br>Accents of different font	Clear
Top box side at 2 locations - Degree sign (o) superscript size	Smaller	Larger	Medium
Bottom box side German text	'Unter 30°C lagem' spelling error with substitution of 'm' for 'rn'. 'Lagem' is not a German word	'Unter 30°C lagern' 'Lagern' is German for store	'Unter 30°C lagern'
Bottom box side at 2 locations Degree sign (o) superscript size	Smaller	Larger	Medium
Numbers Upper Right tab<br>114<br>417<br>891	Wider space between top 2 lines 114 & 417. Text not clear	Narrower space between top 2 lines 114 & 417. Text clear	Equal spacing<br>091<br>400<br>878
Number Upper Left tab	'15'	'17'	'6'

Text Middle Right tab	'76832<br>**dfes'**	'76832<br>**dfes'**	'74802<br>**dfes'**
Middle Right Tab	Coloured bars 8 mm wide and in order:<br>Black<br>Red<br>Pink<br>Black<br><br>No gap between coloured bars and packet edge	Coloured bars 3 mm wide and in order:<br>Black<br>Pink<br>Yellow<br>Black<br><br>Gap between coloured bars and packet edge	Coloured bars 6mm wide and in order:<br>Yellow<br>Red<br>Black<br><br><br>No gap
Bottom Left Tab	Coloured bars 8.5 mm wide	Coloured bars 5.0 mm wide	Coloured bars 6.0 mm wide
LOT	M1200	X0089	X0124
EXP	05 2011	08 2010	07 2007
MFD	01 2009	07 2008	08 2005
NAFDAC Reg. No.	04-3275	04-3275	Not present
**Blisterpack**			
Foil texture and reflectance	Brighter	Duller	Brighter
Blisterpack dimensions/mm	39 x 92	39 x 94	39.5 x 98
Tablets per blisterpack	4 pairs of yellow tablets, embossed with score<br>'N | C'<br>and 'CG' on reverse	4 pairs of yellow tablets, embossed with score<br>'N | C'<br>and 'CG' on reverse	4 pairs of yellow tablets, embossed with score<br>'N | C'<br>and 'CG' on reverse
Printed Text	Different font, bolder font<br>'Coartem 20/120'	Different font, less bold font<br>'Coartem 20/120'	'Coartem 20/120'
Batch number, Mfg Date, Expiry Date	Nil	? X0088 EXP - ??-2-10<br>smudged	X0124 EXP 07 2007
Blister corners	angled	curved	curved
Tablet diameter/thickness mm	9.25/3.54	9.16/3.25	9.12 (9.10-9.13)/ 3.21 (3.21-3.22) [3]
Tablet colour (on CG side) RBG %	69/57/27	54/47/15	99.7/95.1/68.9
**Leaflet**			
Dimensions/mm	145 x 585	141 x 588	148 x 594
Paper colour	White	Creamier	Creamier
Blank paper RGB %	90.5/89.8/94.2	95.0/91.3/92.4	99.4/98.6/99.7
